# A Nanofiber Mat With Dual Bioactive Components and a Biomimetic Matrix Structure for Improving Osteogenesis Effect

**DOI:** 10.3389/fchem.2021.740191

**Published:** 2021-10-29

**Authors:** Yadi Han, Xiaofeng Shen, Sihao Chen, Xiuhui Wang, Juan Du, Tonghe Zhu

**Affiliations:** ^1^ Frontier Institute of Medical & Pharmaceutical Science and Technology, College of Chemistry and Chemical Engineering, Shanghai University of Engineering Science, Shanghai, China; ^2^ Department of Orthopedics, Suzhou TCM Hospital Affiliated to Nanjing University of Chinese Medicine, Suzhou, China; ^3^ Institute of Translational Medicine, Shanghai University, Shanghai, China

**Keywords:** bone tissue injure, nanofiber mat, bioactive components, recognition sites, massive bone tissue regeneration

## Abstract

The challenge of effectively regenerating bone tissue through tissue engineering technology is that most tissue engineering scaffolds cannot imitate the three-dimensional structure and function of the natural extracellular matrix. Herein, we have prepared the poly(L-lactic acid)–based dual bioactive component reinforced nanofiber mats which were named as poly(L-lactic acid)/bovine serum albumin/nanohydroxyapatite (PLLA/BSA/nHAp) with dual bioactive components by combining homogeneous blending and electrospinning technology. The results showed that these nanofiber mats had sufficient mechanical properties and a porous structure suitable for cell growth and migration. Furthermore, the results of cell experiments *in vitro* showed that PLLA/BSA/nHAp composite nanofiber mat could preferably stimulate the proliferation of mouse osteoblastic cells (MC3T3 cells) compared with pure PLLA nanofiber mats. Based on these results, the scaffolds developed in this study are considered to have a great potential to be adhibited as bone repair materials.

## Introduction

Bone defects, accounting for about 50% of surgical operations, caused by various reasons are very prevalent in clinical surgery (Saravanan et al., 2019; Yuan et al., 2020; Wang et al., 2021). Treatment of complex bone defects with irregular shapes or at the interfacial zone with soft tissues, such as cartilage-to-bone, tendon-to-bone, and ligament-to-bone insertion sites remains a clinical challenge (Wang et al., 2019a; Huang et al., 2020). Autograft remains the gold standard for bone repair (Damien and Parsons, 2010; Zimmermann and Moghaddam, 2011). However, it has two fatal flaws limited in clinical practice, namely, significant donor site morbidity and poor capability for machining to accommodate irregular defects. Additionally, autogenous bone transplantation raises significant concerns about the spread of disease and immune responses (Chen et al., 2016; Ye et al., 2019). With the intersection of orthopedic surgery and tissue engineering development, bone substitutes such as ceramics and bone cement have been universally used in clinical practice. However, these materials or scaffolds are usually a three-dimensional structure mismatched with host cells, which may inhibit cell growth, vascularization, and bone regeneration (Jakus et al., 2016; Zhu et al., 2018; Wang et al., 2019b). It is necessary to develop bone tissue engineering scaffolds that are capable of effectively replacing and regenerating fragmented large bulk bone tissue without inducing complications (Oryan et al., 2014; Wang et al., 2019c).

The hybrid fiber material, which is synthesized by electrospinning, possesses the characteristics of enhanced biocompatibility, high porosity, good mechanical properties, large specific surface area, and adjustable hydrophilicity. The micro-/nanofibers prepared by electrospinning can theoretically mimic the three-dimensional structure of an extracellular matrix (ECM), thereby facilitating the proliferation and migration of related cells in the tissue (Li et al., 2014; Rezk et al., 2018a; Aoki et al., 2020). In recent years, new technologies based on electrospinning have been applied to the fabrication of three-dimensional (3D) nanofibrous scaffolds. In these works, fabricated 3D scaffolds derived from electrospun nanofibers have been used for bone tissue engineering. Satpathy *et al.* reported a nanohydroxyapatite (nHAp) nanoparticle-doped electrospun polyvinyl alcohol (PVA)–chitosan composite nanofibrous mat, which was successfully fabricated with improved performance for the potential application as a bone tissue regeneration material. The author claims that the incorporation of HAp nanoparticles improves the biocompatibility as well as bioactivity of PVA–chitosan composite scaffolds for osteoblast, and such composite scaffolds may serve as a good template for bone tissue engineering (Satpathy et al., 2019). Ye *et al.* prepared a bone scaffold (nanohydroxyapatite/PLLA/gelatin (nHA/PLA/GEL) 3D nanofibrous scaffolds) based on combining electrospinning, homogenizing, freeze-drying, and thermal crosslinking techniques. This research concluded that the combined use of nHA and the BMP-2-derived peptides synergistically and significantly promoted BMSC osteogenic differentiation *in vitro* and bone regeneration in a rat cranial bone defect model (Ye et al., 2019).

Inorganic osteogenic nanoparticles have been extensively synthesized for a bone regeneration scaffold due to its easily modified surface (Liu et al., 2016). Sokolova et al. reported a porous scaffold, which is composed of poly(lactide–coglycolide) (PLGA) and nanohydroxyapatite (nHAp) for bone substitution. All authors in this research alleged that it is expected to enhance bone growth around an implanted scaffold or inside a scaffold for tissue engineering (Sokolova et al., 2020). Bauer et al. synthesized biphasic nHAp and whitlockite composite scaffolds with different ratios by changing the content of Mg^2+^ ions for strengthening osteogenic differentiation of human mesenchymal stem cells (hMSCs) (Bauer et al., 2020). Chen et al. prepared porous hydroxyapatite composite scaffolds through 3D bioprinting for bone tissue engineering (Chen et al., 2019). These reported scaffolds with different proportions of inorganic osteogenic nanoparticles had porous structures for facilitating cell proliferation and migration, but the matrix materials used do not have the sufficient ability to induce effective osteogenesis, which is mainly due to the lack of strong bioactive sites that recognize the host cytomembranes.

Bovine serum protein (BSA) is a globulin in bovine serum, mainly composed of proteins, peptides, hormones, etc., and it has abundant cell recognition sites to promote cell adhesion and proliferation (Li et al., 2017; Liu et al., 2020). As in previous research, a linear gradient of active protein was readily loaded into uniaxially aligned nanofibers by backfilling the active protein onto the bare regions by a graded mask of BSA (Tanes et al., 2017). Additionally, Wu et al. demonstrated a simple and general strategy by first creating a graded mask of BSA on nanofibers; it is beneficial to improve the biocompatibility of the material (Wu et al., 2018). However, they added the different gradient growth factors for the purpose of improving tissue regeneration, but the release of growth factors was difficult to control.

Poly(L-lactic acid) (PLLA) is a biodegradable material that is often used for tissue regeneration, whose degradation cycle and mechanical properties matched the growth of osteoblasts (Shim et al., 2010; Schofer et al., 2012; Qi et al., 2016; Wang et al., 2016). Beyond this, PLLA can be blended with natural active materials or inorganic particulate matter for electrospinning (Shim et al., 2010; Rajzer et al., 2018; Ye et al., 2019). However, the acidic degradation products of pure PLLA can inhibit osteoblasts and promote the growth of osteoclasts, which may have a negative effect on bone regeneration. Based on this, we believe that, by combining these bioactive factors, its drawbacks can be modified and their advantages will be magnified. The synergistic effect of these three substances will provide a biomimetic microenvironment for the growth of osteoblasts. Therefore, we will attempt to use PLLA as the base framework material, adding bioactive BSA and polyhydric nHAp dual components to neutralize the acid product as well as improve the osteogenesis effect.

## Materials and Methods

### Materials

Poly(L-lactic acid) (PLLA, M_w_ = 125,000 g/mol) was purchased from Shenzhen MaiQi Biomaterials Co., Ltd. (Shenzhen, China). BSA (M_w_ = 66,446 g/mol, isoelectric point = 4.7) was purchased from Shanghai Titan Scientific Co., Ltd. (Shanghai, China). Nanohydroxyapatite (nHAp) was purchased from Shanghai Aladdin Biochemical Technology Co., Ltd. (Shanghai, China). 1,1,1,3,3,3-Hexafluoro-2-propanol (HFIP) was purchased from Shanghai Darui Fine Chemical Co., Ltd. (Shanghai, China). MC3T3 cells were obtained from Shanghai Institute of Biochemistry and Cell Biology (SIBCB, CAS, China). All culture media and reagents were purchased from Invitrogen and Sigma-Aldrich (St. Louis, MO) unless stated otherwise. All chemicals used in the experiments are analytical grade reagents and used without further purification.

### Fabrication of Nanofiber Mats

The nanofibers mat was prepared by the electrospinning technology. In brief, 0.8 g PLLA and 0.08 g BSA was dissolved into 10 ml HFIP under vigorous stirring at room temperature for 24 h. Then, the HFIP solution with a uniform concentration of 1% nHAp was added into the solution for preparing PLLA/BSA/nHAp electrospinning solution. After 48 h of intense magnetic stirring, the well-mixed solution was drawn into a plastic syringe, followed by connecting it with the DC voltage and sprayed at a feeding rate of 1.0 ml/h through a metal needle with an inner diameter of 0.8 mm at the same time. The nanofiber mat is collected on a rectangular grounded metal plate covered with an aluminum foil. The voltage and receiving distance between the needle and the metal plate was set at 10 kV and 15 cm, respectively. All processes were carried out at about 50 ± 5% relative humidity. The collected nanofibers mat was first put into a fume hood for self-volatilizing for 48 h to remove residual HFIP and then put in a vacuum drying oven for 24 h at a constant temperature for subsequent characterization and testing.

### Characterization of Nanofibers and Nanofiber Mats

A scanning electron microscope (SEM, Phenom XL, Netherlands) was used to test the microscopic morphology of the nanofibers after spraying gold. The diameter distribution of nanofibers from the SEM fiber images can be measured by using ImageJ software (National Institutes of Health, MD, United States). To see the structure of single fiber, the fiber was further measured by transmission electron field electron microscopy (TEM, JEOL JEM-2100).

The apparent water contact angle (WCA) was measured three times for each sample using a contact angle instrument (OCA40, DataPhysics, Germany) when the deionized water droplet was stable at room temperature. Both WCA values of the left and right sides were measured, and an average value was applied.

The porosity of nanofiber mats was measured by the ethanol immersion method as previously described using the reported formula (Zhu et al., 2017; Du et al., 2019). In brief, 5 ml (*V*_*1*_) ethanol was first filled in a measuring cylinder, and then nanofibrous mats (*n* = 4) were immersed in ethanol for 10 min, respectively. The resulting volume of ethanol was recorded as *V*_*2*_, and the residual volume of ethanol in a measuring cylinder after removing the wet nanofibrous mats was *V*_*3*_. The porosity (p) was calculated according to the following formula:

$$p\left(\%\right)=\left(V_{1}-V_{3}\right)/\left(V_{2}-V_{3}\right).$$ (1)

The apparent density (*ρ*_a_) was calculated by *ρ*_a_ = *m*/*v,* where *m* is the mass and *v* stands for the volume of the samples (Du et al., 2019).

The structure and properties of the nanofiber mat were analyzed by Fourier transform infrared spectroscopy (FTIR, Avatar 380, United States) in the range of 400–4,000 cm^−1^ wavenumbers at a resolution of 2 cm^−1^. Using X-ray diffraction (XRD, Tokyo, Japan) under the condition of 40 KV tested the crystal structure of the nanofiber mat, sample scanning was performed at a 2*θ* angle (5°–80°). STAPT-1000 instruments (Linseis, German) were used to evaluate the thermal properties andthe thermal stability of nanofiber mats. In brief, the samples were heated from 25°C to 800°C under a flow of nitrogen (10 ml/min), and the thermogravimetric analysis (TGA) curves were measured at a heating rate of 10°C/min.

The stress–strain curves and Young’s modulus of the nanofiber mats were tested in dry conditions using a general test machine (HY-940FS, Shanghai Hengyu Instrument Co., Ltd., China) (Hong et al., 2011; Zheng et al., 2013). Before the measurement, three sample strips were randomly cut from nanofiber mat for the tensile test to get an average result. The tensile speed of nanofibers mats was 5 mm/min before the sample fracture.

### Cell Culture With Nanofiber Mats

MC3T3 cells were grown in a carbon dioxide incubator at 37°C and 5% CO_2_. A Dulbecco’s modified eagle medium (DMEM) containing 10% fetal bovine serum and 1% penicillin–streptomycin solution was used. Nanofiber mats were prepared on glass cover plates and placed at the bottom of 24-well plates, respectively. The surface of the sample was fixed by a stainless steel ring. Before inoculation, the stainless steel ring, glass cover plates, and nanofiber mat were sterilized with 75% ethanol for 12 h and then soaked in a culture medium to ensure a uniform distribution on the nanofibers mat. Then, MC3T3 cells with a certain density (1.0 × 10^4^/well) were incubated on the nanofiber mat for cell adhesion and proliferation, and the medium was changed every 3 days. After MC3T3 cells were cultured in a 24-well plate for 1, 4, and 7 days, the cell viability was determined by the CCK-8 method. Fluorescence microscopy (Nikon, Japan) and SEM were used to observe the cell adhesion of nanofibers mats. For the immunofluorescence observation, the samples were fixed with 4% paraformaldehyde, following staining with rhodamine-conjugated phalloidin and DAPI. Finally, the residual dye of actin and nuclei of the cells were washed with PBS.

### Western Blot Testing

For the determination of osteogenic-associated gene expression level of MC3T3 cells which were cocultured with different nanofiber mats, MC3T3 cells (1 × 10^5^ cells/well) were cultured in a 6-well plate for 1, 4, and 7 days. At a period of 1, 4, and 7 days of culture, sodium dodecyl sulfate polyacrylamide gel electrophoresis was used for the protein extraction, which was then moved onto a polyvinylidene difluoride membrane surface (Merck Millipore, German). The membranes were then treated with 5% bovine serum albumin (BSA) and then incubated with monoclonal antibodies for RUNX2, OCN, and actin at 4°C overnight. The samples were then incubated with HRP-conjugated secondary goat anti-rabbit antibody (RT, 2 h), followed by the quantitative analysis using Amersham ECL Plus reagents on an Image Quant LAS 4000 (GE, United States).

### Statistical Analysis

The results of all our work were expressed as mean standard deviation (SD) one-way ANOVA for data analysis, and Origin Pro 9.5 for the Tukey’s test for specificity difference assessment with a significant level set at 0.05.

## Results and Discussion

**FIGURE 1:**
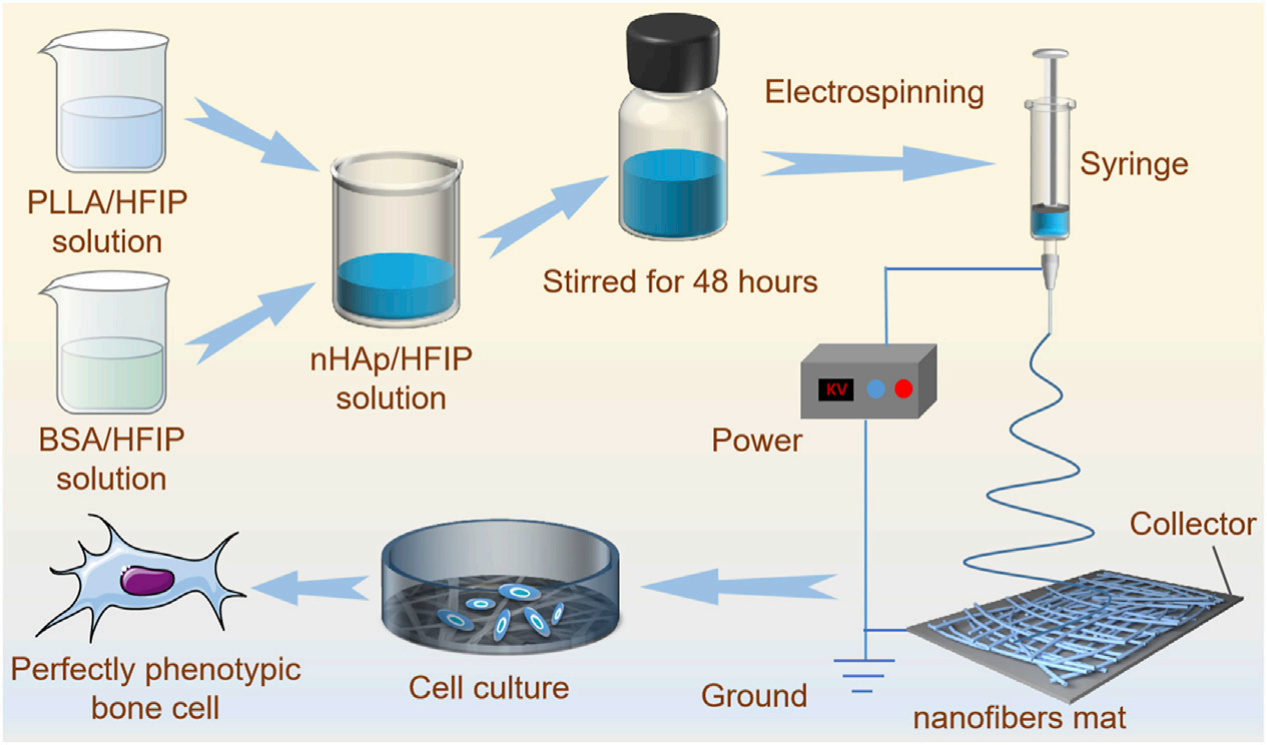
The schematic diagram of PLLA/BSA/nHAp nanofiber mat preparation by electrospinning and mechanisms of regulating bone cellular phenotype by nanofibers.

A simple and effective strategy to regulate the preparation and construction of functional bone regeneration induced nanofibers mats with dual bioactive components via homogeneous blends combined with the electrospinning technology is proposed. Figure 1 shows the fabrication process and formation mechanism of the functional scaffold with a three-dimensional structure. We prepared a uniformly dispersed mixed solution by separately dissolving the components and then blending them. This solution is easier to electrospin to form regular fibers than a solution which is prepared by “one-pot blending.” The biodegradable PLLA as a frame material for mechanical support, as well as bioactive polymer BSA from a natural life source with osteogenic induction activity, and nHAp nanoparticles were combined together by electrospinning to promote bone regeneration.

### Apparent Morphology and Microstructure of Prepared Nanofibers

Nanofiber-based scaffolds are very suitable material for bone repairing due to its three-dimensional structure with a biomimetic extracellular matrix, which can enhance differentiation of osteoblasts and mineralization due to their ECM-like structures (Srouji et al., 2011; Cheng et al., 2018; Kouhi et al., 2019). Bone tissue is generally composed of inorganic salts (e.g., HAp), organic fibrous matrices (collagen I), and cells. As a result, bone repair requires the application of inorganic materials to pack the defect and collagen membrane to inhibit the rapid growth of nearby soft tissues (Feng et al., 2019). Therefore, by incorporating the osteogenic active substance into an electrospun scaffold *via* the electrospinning technology, it can promote the osteogenic differentiation and bone healing.

**FIGURE 2:**
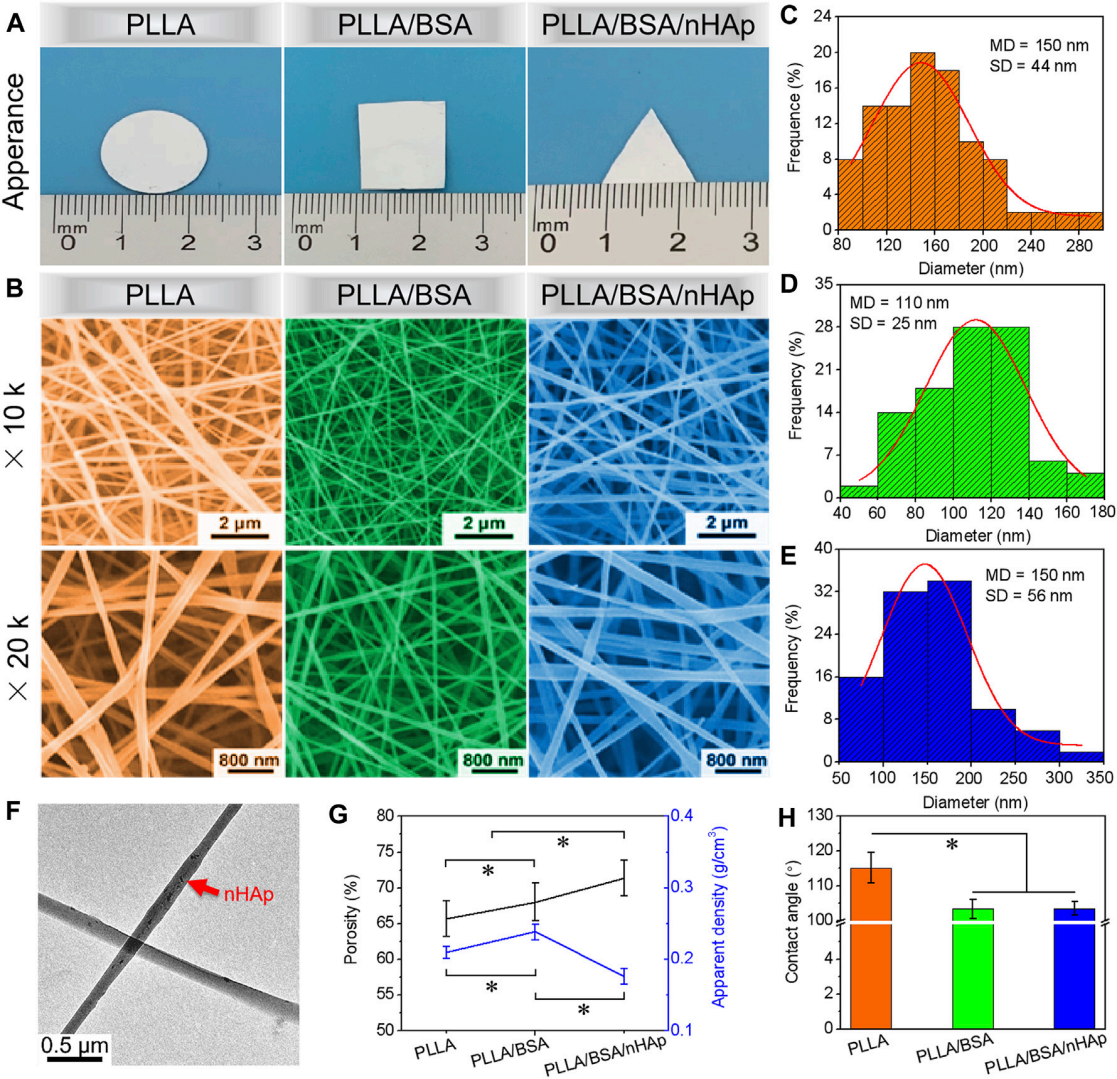
**(A)** The appearance images and **(B)** SEM images at different multiples of prepared PLLA, PLLA/BSA, and PLLA/BSA/nHAp nanofibers. **(C–E)** Normal distribution of PLLA, PLLA/BSA, and PLLA/BSA/nHAp nanofibers, respectively. **(F)** TEM image of PLLA/BSA/nHAp nanofiber. **(G)** Porosity and apparent density. **(H)** Water contact angle of PLLA, PLLA/BSA, and PLLA/BSA/nHAp nanofibers mats, respectively. (*p < 0.05).

In this study, three nanofibers mats, namely, PLLA, PLLA/BSA, and PLLA/BSA/nHAp nanofiber mats were prepared as an induction carrier for bone regeneration. PLLA, PLLA/BSA, and PLLA/BSA/nHAp nanofiber mats can also be easily processed into any shape and appearance as shown in Figure 2A. The three-dimensional structure of nanofiber mats is closely related to the cell phenotype. The microstructure of nanofiber mats is not only related to the electrospinning technological parameter, such as voltage, solution concentration, and receiving distance, but also related to the dispersibility of nHAp nanoparticles in polymer materials. Therefore, we have electrospun PLLA, PLLA/BSA, and PLLA/BSA/nHAp—three kinds of fibers—under the best electrospinning by adjusting parameters and tested its morphology by SEM. As shown in Figures 2B–E, all the nanofibers’ surface renders an interconnected porous network structure by SEM, and the diameter of the nanofibers remains relatively well with ImageJ analysis software. In addition to this, we found that the PLLA, PLLA/BSA, and PLLA/BSA/nHAp nanofibers diameter, respectively, were 0.150 ± 0.044 μm, 0.110 ± 0.025 μm, and 0.150 ± 0.056 μm (Figures 2C–2E). Compared with pure PLLA nanofiber mats, the reduced diameter of the PLLA/BSA nanofibers may be related to the presence of positive charge in BSA, which neutralized with the negative charge output by the voltage generator during the electrospinning. On the other hand, the mercapto group in BSA is very active, which can bind to a variety of small molecules and enhance the tractive force of the droplet surface in the process of electrospinning. What is noteworthy is that, compared with the PLLA/BSA nanofiber mats, PLLA/BSA/nHAp nanofibers mat has a larger diameter, which may be due to the addition of the uniform dispersion of nHAp particles as in Figure 2F (red arrow). Although nHAp is uniformly mixed in the electrospinning solution, it also increased the viscosity of the mixed solution, so the diameter of PLLA/BSA/nHAp nanofibers is relatively thicker than that of PLLA/BSA nanofibers.

It is reported that a higher porosity could provide cells with more space for attachment and proliferation and also improve nutrition transport (Menon et al., 2015). As shown in Figure 2G, among PLLA, PLLA/BSA, and PLLA/BSA/nHAp nanofiber mats, PLLA/BSA/nHAp nanofiber mat has the largest porosity and the smallest apparent density. The porosity and apparent density of prepared nanofiber mats are related to the fiber diameter and the three-dimensional structure of mats. Although PLLA/BSA/nHAp nanofiber mat and PLLA nanofiber mat have the same statistical average fiber diameter, the morphology of PLLA/BSA/nHAp nanofiber is not regular due to the dispersion of nanoparticles in a fiber matrix.

The hydrophilicity of biomaterials also plays an extremely important role in tissue engineering applications (Yu et al., 2020). The wetting property of the fiber mats is not only related to mats’ three-dimensional structure but also related to the physical and chemical properties of the mats material itself. PLLA has weak hydrophilicity and no natural cell recognition site, which leads to a poor cell affinity. However, the natural material BSA has a lot of natural cells recognition sites and preferable cellular affinity, which could be the high potential scaffold material of bone tissue engineering. Herein, the water contact angle was used to evaluate the surface hydrophilicity of prepared nanofiber mats. As illustrated in Figure 2H, the dynamic change of the water contact angle was measured to evaluate the hydrophilicity of mats. The obtained results demonstrated that the water contact angle of PLLA/BSA nanofiber mat decreased compared with the PLLA nanofibers mat. This can be attributed to the hydrophobic property of PLLA. Compared with the PLLA/BSA nanofibers mat, the hydrophilicity of PLLA/BSA/nHAp nanofibers mat decreased again, indicating that nHAp further enhanced the hydrophilicity of nanofibers mat due to its rough fibrous surface structure, which may help the nanofiber mat to recruit more cells on the surface of mats.

### Chemical Structure and Characteristics

**FIGURE 3:**
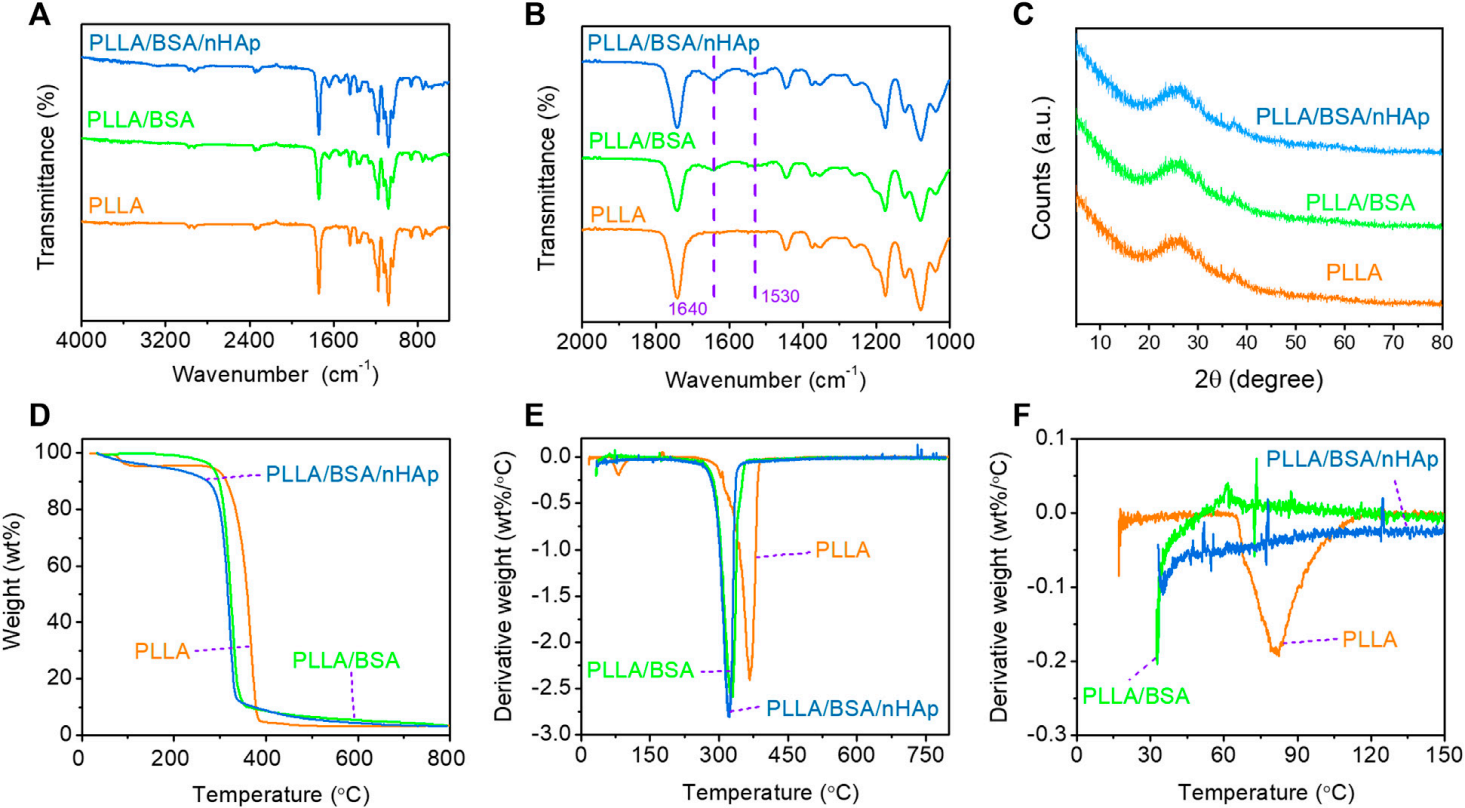
Chemical properties of nanofibers mat include the following: **(A, B)** FTIR spectra curves of 4,000–400 cm^−1^ range and 2000–1,000 cm^−1^ range; **(C)** XRD with 5–80°range; **(D)** TGA curves with 50–800°C range, and **(E, F)** DTG curves with 0–800°C and 0–150°C range.

The ATR-FTIR spectrum identifies materials and their interactions by determining chemical bonds (Holm et al., 2007). According to Figures 3A,B, it can be seen from the infrared spectrum of the PLLA nanofiber mat that there was no obvious absorption peak above 3,000 cm^−1^, which indicates that PLLA basically contains no hydroxyl and carboxyl groups. The absorption peak at 1746.09 cm^−1^ represented the stretching vibration of ester carbonyl in PLLA. The peak around 2,977.08 cm^−1^ was the -C-H- group stretching vibration of PLLA. The absorption peak at the wavelength of 1,085.34 cm^−1^ represented the stretching vibration absorption of -C-O- in PLLA. Compared with a pure PLLA nanofiber mat, the characteristic peak of both PLLA/BSA and PLLA/BSA/nHAp nanofiber mats appeared around 1,530–1,640 cm^−1^, which may due to the presence of α-helical proteins in BSA (Patel et al., 2019). The comparison of PLLA/BSA and PLLA/BSA/nHAp nanofibers mats showed no significant change in the peak value, indicating that the addition of BSA and nHAp nanoparticles had little effect on the structure of polymer PLLA, and it also indicates that the three substances are closely bound together by an intermolecular force or hydrogen bonding.

The crystal structures of the PLLA nanofibers mat and composite nanofibers mats were accurately characterized by XRD (Figure 3C). The inorganic phase composition of the PLLA/BSA/nHAp nanofibers mat was studied by the XRD analysis. As previously reported, PLLA polymer is a semicrystalline material that can be identified by XRD at specific peaks at 21.5° and 29.5°. Compared with the PLLA nanofiber mat, the XRD patterns of the PLLA/BSA/nHAp nanofibers mat had little changes in the crystal structure, due to the excellent physical mixing between apatite nanoparticles and PLLA/BSA matrix.

The thermogravimetric analysis reveals the change of material mass with the increase of temperature and time to evaluate the thermal stability of the material (Zhu et al., 2017). The principle of TGA detection is to measure the residue after the thermal degradation of the sample and evaluate the thermal stability of the sample. TGA curves of nanofibers mats are shown in Figure 3D. We could simply analyze that the weight loss at 250–300°C was caused by the thermal decomposition of PLLA. In addition, there was only one turning point on the thermal degradation curves of the three materials, and the initial degradation temperature was between 250 and 300°C, which indicated that the addition of BSA and nHAp did not significantly change the thermal stability of the composites compared with the PLLA nanofiber mat. As shown in Figures 3E,F, the peak thermal decomposition temperatures of different components are different, and it represents the maximum rate of weight loss that occurs. The DTG curve reveals that the initial temperature of the thermal degradation of nanofibers decreases with the addition of BSA, and this is due to BSA, which is a natural active ingredient with poor thermal stability; the heating process may lead to the aggravation of an intermolecular motion in the nanofiber mat. Therefore, the maximum thermal decomposition rate corresponding to the temperature of composite nanofibers mats was reduced by further introducing BSA.

### Mechanical Properties

**FIGURE 4:**
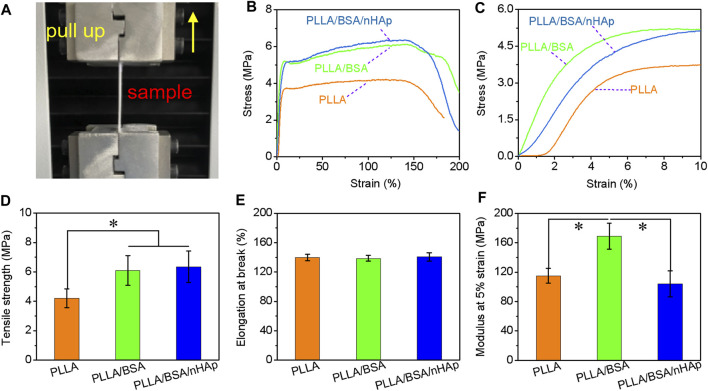
Mechanical properties of nanofibers mats: **(A)** photographs of fracture of mat strips; **(B)** stress-strain curves from begin to break, and **(C)** stress–strain curves from begin to 10% strain; **(D)** tensile strength, **(E)** elongation at break, and **(F)** modulus at 5% strain of PLLA, PLLA/BSA, and PLLA/BSA/nHAp nanofibers mat under a dry situation. (*p < 0.05).

Suitable mechanical properties similar to the bone tissue are beneficial for bone tissue repairing via a specific cell phenotype (Matinfar et al., 2019; Zhu et al., 2019). In this study, the radial stress–strain curves, modulus at 5% strain, tensile strength, and elongation at break of PLLA, PLLA/BSA, and PLLA/BSA/nHAp nanofiber mats under dry state tested by a unidirectional stretching machine were used to evaluate mats’ mechanical properties (Figure 4A). As shown in Figures 4B,C, it was obvious that all the nanofiber mats showed a regular curve with a quick increase. Additionally, we can see the tensile strength of the PLLA/BSA nanofibers mat was strikingly higher than the pure PLLA nanofiber mat. This phenomenon was attributed to the coexistence of BSA and PLLA, which leads to the enhancement of the intermolecular force of electrospun solution. Much more than this, the decrease of fiber diameter and the increase of nanofiber mat density can also lead to an increase of tensile strength and initial modulus. Additionally, the introduction of BSA also further improved the tensile strength, and this may be related to the hydrogen bond interface and a serious entanglement occurred between the molecular chains of BSA and PLLA components (Figure 4D). The addition of BSA and nHAp did not significantly change the elongation at break of the scaffold, possibly due to nHAp and BSA evenly dispersed in the fibers (Figure 4E). The modulus test at 5% strain results showed that, with the addition of BSA, the modulus of the PLLA nanofiber mat was increased. But when nHAp is continued to be added, the modulus of the PLLA/BSA nanofiber mat was decreased, which is the appearance of nHAp caused by a more serious nHAp–PLLA or nHAp–BSA interface bonding.

### Biocompatibility and Osteogenic Effect in *In Vitro*

**FIGURE 5:**
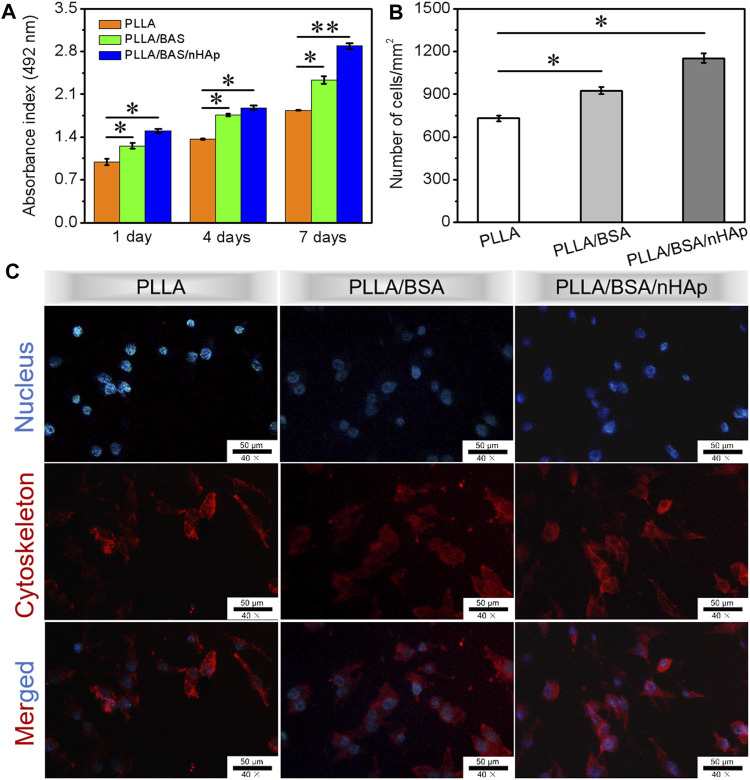
Biocompatibility tests results: **(A)** CCK-8 assay of the proliferation viability of MC3T3 cultured for 1, 4, and 7 days. **(B)** Number of MC3T3 cells per mm^2^ after culture for 7 days. **(C)** DAPI (blue)/rhodamine-conjugated phalloidin (red) staining assay after 4 days of culture. (**p < 0.01 and *p < 0.05).

As we know, the micro-/nanofibers can mimic the three-dimensional structure of the extracellular matrix, which will provide a matrix for cell adhesion and proliferation. Ensuring cell survival *in vitro* is one of the key techniques for assessing the biocompatibility of scaffolds (Rezk et al., 2018b; Du et al., 2018; Rather et al., 2020). As one of the most important elements in bone tissue engineering, MC3T3 was selected in this study to evaluate their proliferation characteristics on nanofiber mat. The proliferation of MC3T3 incubated on the surface of nanofibers mat was analyzed by using the CCK-8 assay after 1, 4, and 7 days (*n* = 3 for each group) of culture. From the histogram in Figure 5A, the results indicated that a slight increase in the proliferation profile on the PLLA/BSA composites nanofiber mat and PLLA/BSA/nHAp nanofibers mat than pure PLLA nanofibers mat was observed after 1 day of culture. The number of MC3T3 cells per mm^2^ after culture for 7 days is shown in Figure 5B. After 7 days of culture, the number of cells on both the PLLA/BSA nanofibers mat and PLLA/BSA/nHAp nanofibers mat was more compared with the PLLA nanofibers mat. The cells showed a good proliferative activity on the surface of the PLLA/BSA/nHAp nanofiber, mat and the biocompatibility of the scaffold was good. The results indicated that PLLA/BSA/nHAp nanofiber mat with a biomimetic extracellular matrix was beneficial to the growth of osteoblasts.

DAPI and rhodamine-conjugated phalloidin were applied to staining nucleus and cytoskeleton of proliferative MC3T3 cells with blue fluorescence and red fluorescence, respectively. The morphology of the cellular activity from fluorescence microscopy images is shown in Figure 5C for day 4 of MC3T3 proliferation. The results indicated that MC3T3 cells cultured on the surfaces of PLLA and PLLA/BSA nanofiber mats presented a similar polygonal shape, while on the surfaces of the PLLA/BSA/nHAp nanofiber mat spread more homogeneously and presented cell-specific pseudopods, suggesting that the addition of nHAp uniformly dispersed in the PLLA/BSA nanofibrous matrix promoted the cell proliferation on nanofiber mat. Therefore, the cells might achieve rapid osteogenic differentiation on PLLA/BSA/nHAp nanofibers mat, indicating the potential application of bone tissue engineering.

**FIGURE 6:**
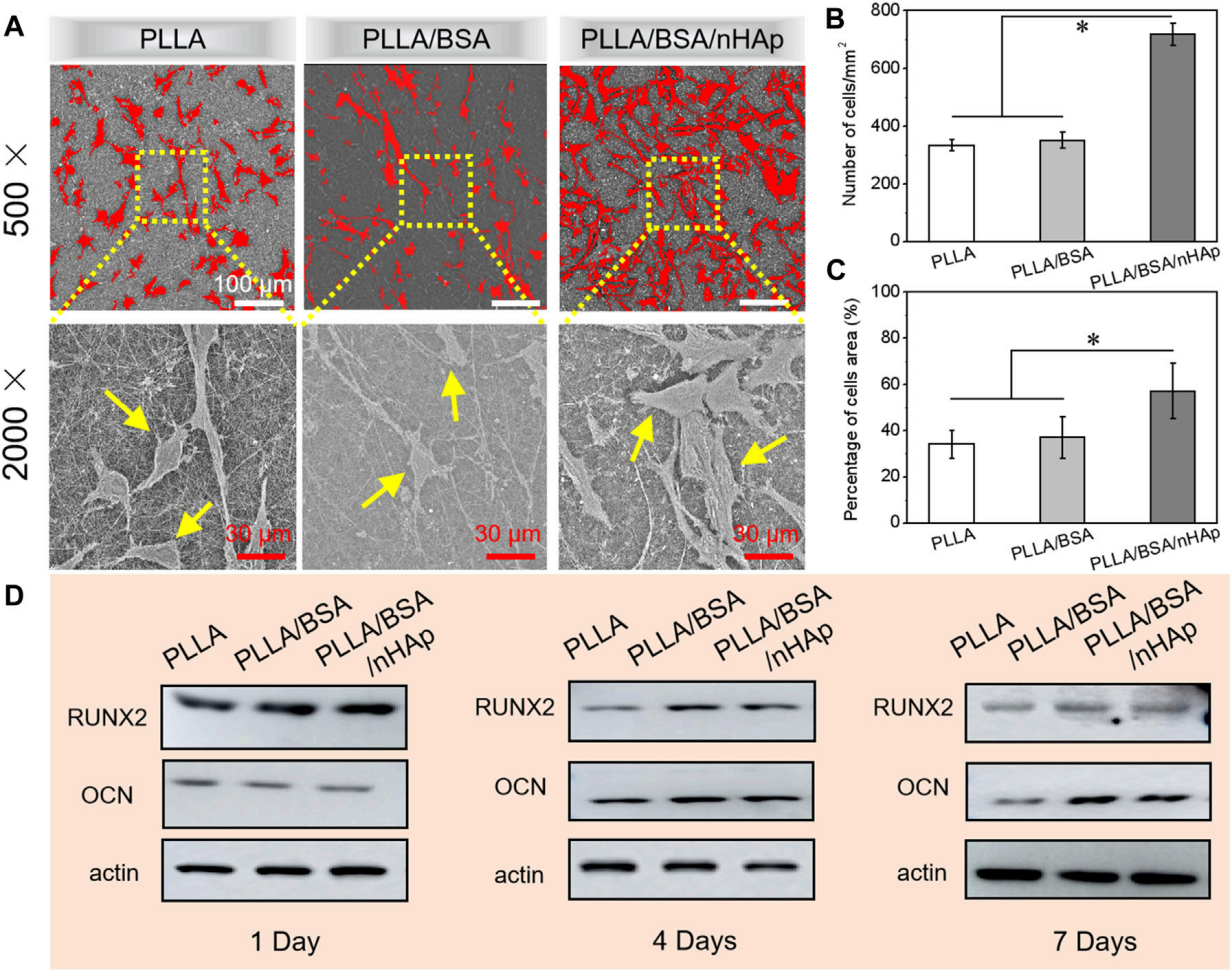
**(A)** SEM images of cells after 4 days of culture *in vitro*. **(B, C)** Number of MC3T3 cells per mm^2^ and percentage of cells area after 4 days’ culture, respectively. **(D)** Western blot results for detecting proteins (RUNX2, OCN, and β-actin) associated with osteogenesis in MC3T3 cultured for 1, 4, and 7 days, respectively (*p < 0.05).

After cells adhere to the surface of nanofibers mats, they entered the stage of rapid proliferation and growth. Therefore, the establishment of cell–cell interaction is essential for the subsequent differentiation and growth of cells. SEM images of MC3T3 cells proliferation after seeding on different nanofiber mats for 4 days are shown in Figure 6A. It can be seen that the MC3T3 was equally deposited onto the surfaces of scaffolds. Moreover, according to the results of the number of MC3T3 cells per mm^2^ and percentage of cell area as in Figures 6B,C, MC3T3 cells on the surfaces of PLLA/BSA/nHAp nanofibers mat displayed spindle or triangular shapes and showed a greater spread property than the other two groups. The results suggested that PLLA/BSA/nHAp composite nanofiber mat could provide the appropriate environment for cell adhesion and proliferation.

The basic principle of western blot is to stain the cells or biological tissue samples treated by gel electrophoresis with specific antibodies and obtain the information of the expression of specific proteins by analyzing the location and depth of staining. The expression of osteogenic proteins (i.e., RUNX2, OCN, and β-actin) in MC3T3 cultured with PLLA nanofiber mat, PLLA/BSA nanofiber mat, and PLLA/BSA/nHAp nanofiber mat for 1 day, 4, and 7 days were qualitatively detected by a western blot assay, respectively. As shown in Figure 6D, the cell cultured with the PLLA/BSA/HAp nanofibers mat was shown obviously increased the protein expression level more than the PLLA and PLLA/BSA nanofiber mat after either 4 days or 7 days. It is worth noticing that the band intensity of PLLA/BSA was the strongest among the three kinds of nanofibers mats, indicating that the osteogenesis-related protein expression levels in the group with BSA were significantly better than pure PLLA nanofibers mat after 4 days. The protein expression also showed a similar trend after 7 days, indicating that the addition of BSA and HAp induced the preferable osteogenesis effect.

## Conclusion

In summary, a nanofiber mat with dual bioactive components and biomimetic matrix structure (PLLA/BSA/nHAp) with improved osteogenesis capability was successfully developed by homogeneous blending, following the electrospinning of poly(L-lactic acid), bovine serum albumin, and nanohydroxyapatite as comonomers in this work. PSM also exhibited salutary surface wettability and matched mechanical properties with bone tissue. A coculture with cells and an osteogenic effect in *in vitro* studies indicated that the migration and protein (RUNX2, OCN, and β-actin) associated with the osteogenesis of osteoblast can prominently speed up by the bioactive nanofibers mat. Meanwhile, the introduction of BSA and nHAp in PLLA nanofibers enables PLLA/BSA/nHAp with the capability of promoting ossification, which substantially facilitates the potential osseous regeneration to repair a large bone defect. Therefore, it is envisioned that PLLA/BSA/nHAp as promising bioactive scaffolds holds a high potential in the applications of bone tissue engineering.

## Data Availability

The raw data supporting the conclusions of this article will be made available by the authors, without undue reservation.
