# Supplementary material for: A Nanofiber Mat With Dual Bioactive Components and a Biomimetic Matrix Structure for Improving Osteogenesis Effect
Source: Front Chem. 2021 Oct 29;9:740191. doi: 10.3389/fchem.2021.740191 (PMC8586446; doi:10.3389/fchem.2021.740191)
Supplement: Supplementary file 1 [file DataSheet1.ZIP › Fig1-schematic diagram.pptx]

## Slide 1
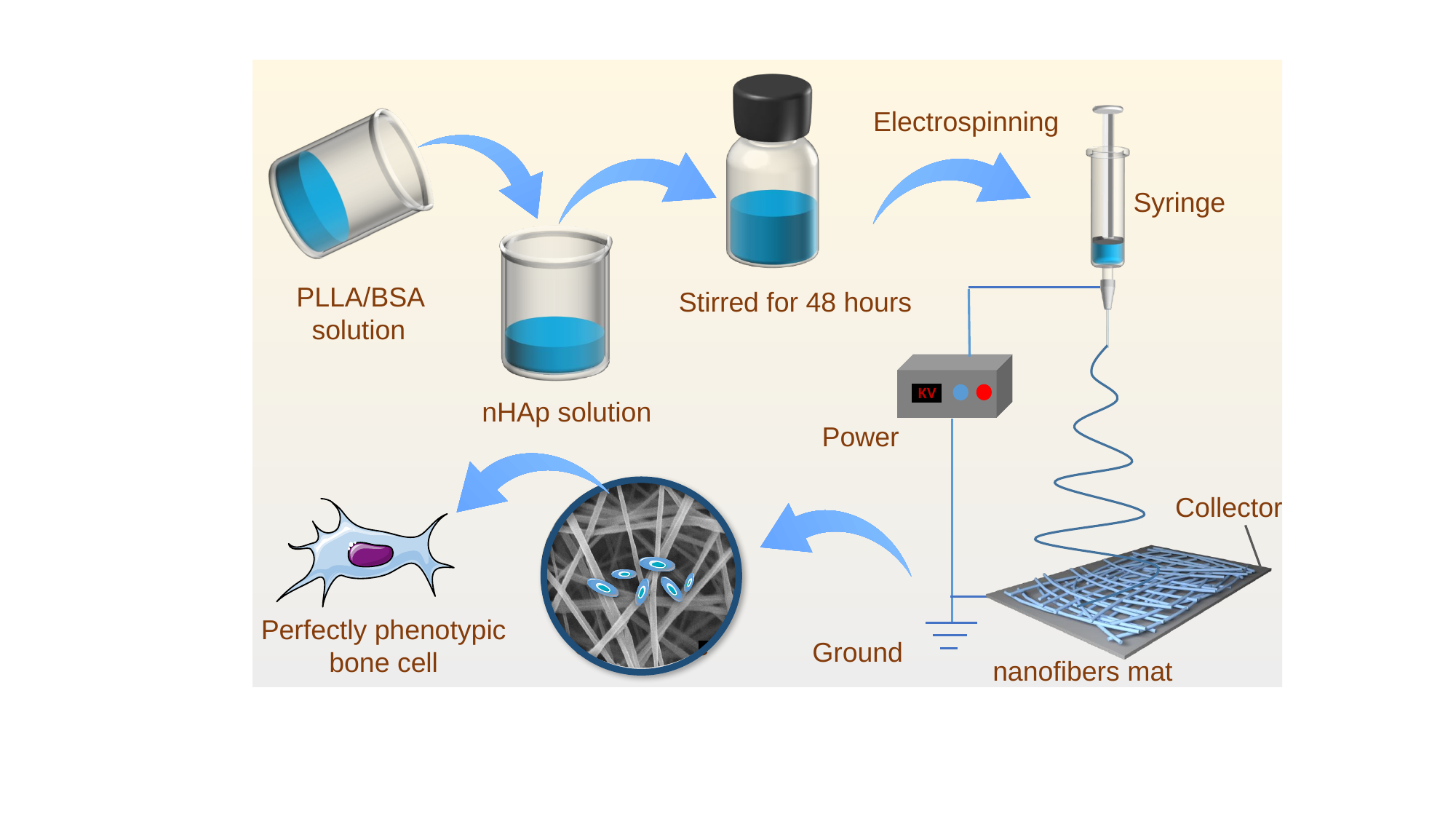

Electrospinning
Syringe
PLLA/BSA
 solution
Stirred for 48 hours
KV
nHAp solution
Power
Collector
Perfectly phenotypic
bone cell
Cell culture
Ground
nanofibers mat

## Slide 2
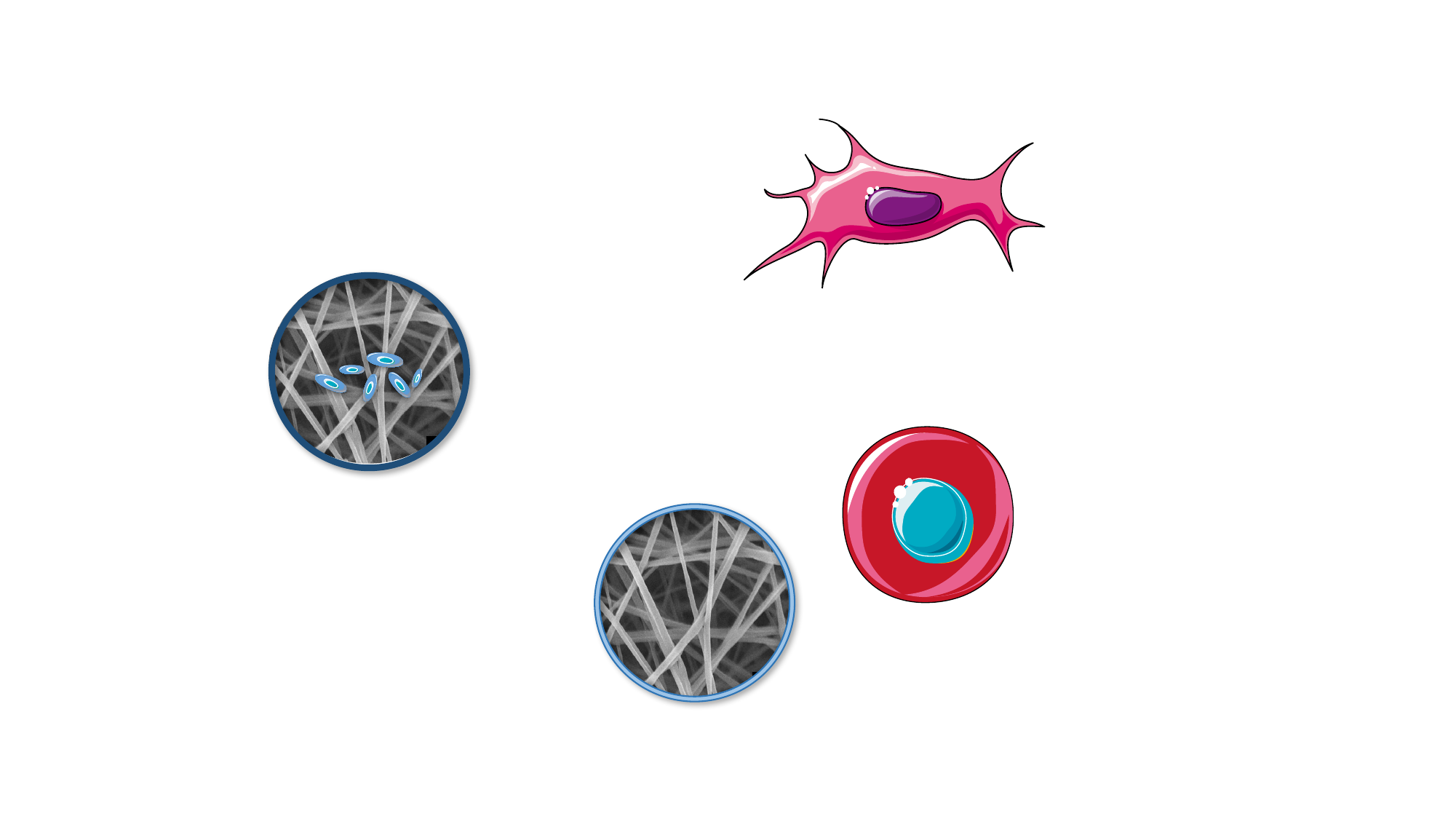

## Slide 3
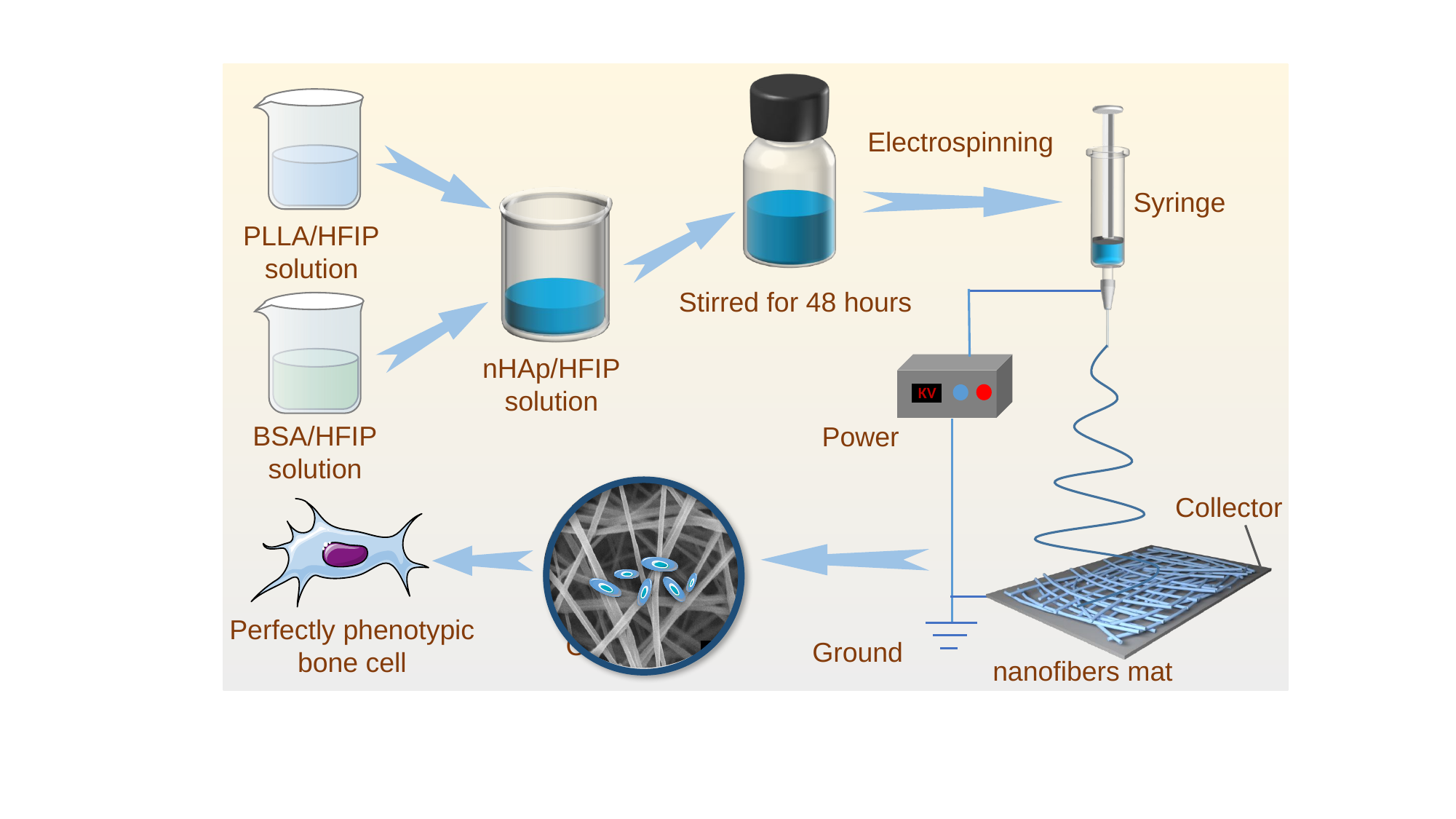

Electrospinning
Syringe
PLLA/HFIP
solution
Stirred for 48 hours
nHAp/HFIP
solution
KV
BSA/HFIP
solution
Power
Collector
Perfectly phenotypic
bone cell
Cell culture
Ground
nanofibers mat
