# Supplementary figures and images for: A Nanofiber Mat With Dual Bioactive Components and a Biomimetic Matrix Structure for Improving Osteogenesis Effect
Source: Front Chem. 2021 Oct 29;9:740191. doi: 10.3389/fchem.2021.740191 (PMC8586446; doi:10.3389/fchem.2021.740191)

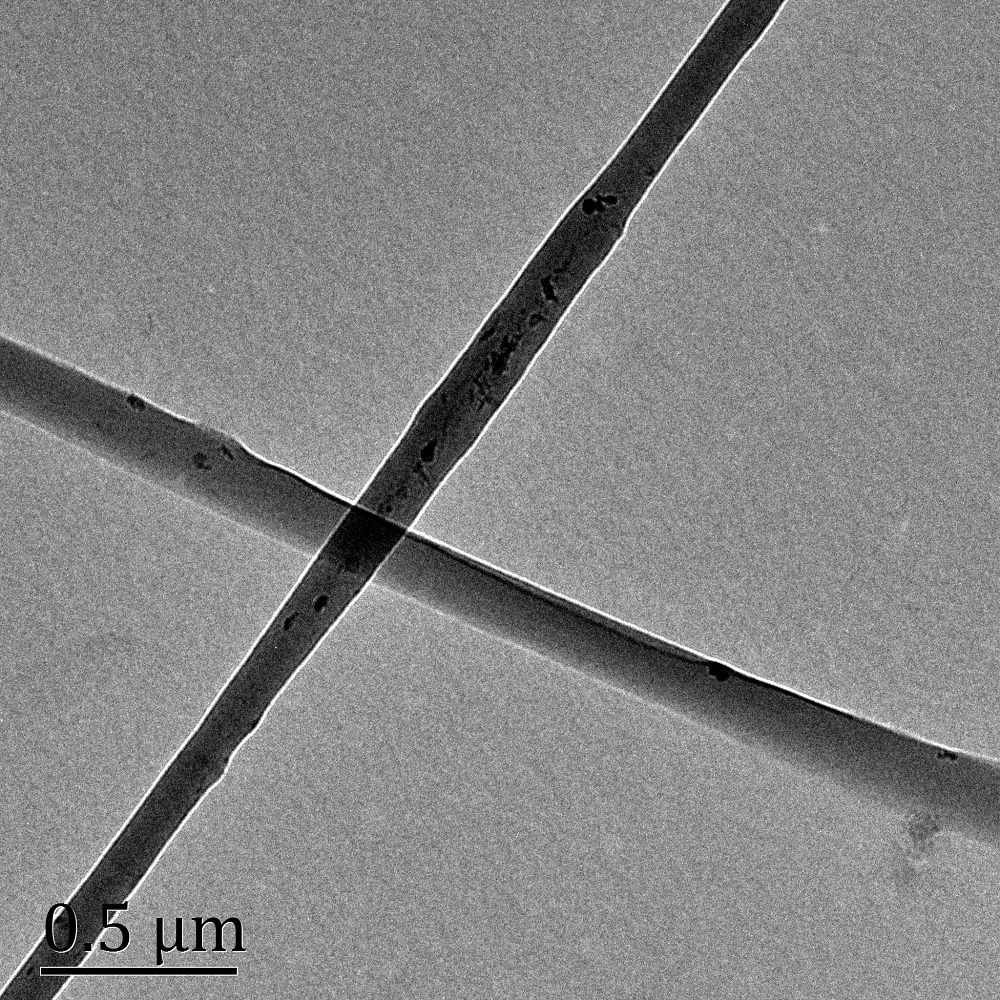

Supplement: Supplementary file 1 [file DataSheet1.ZIP › Fig 2-TEM.tif]

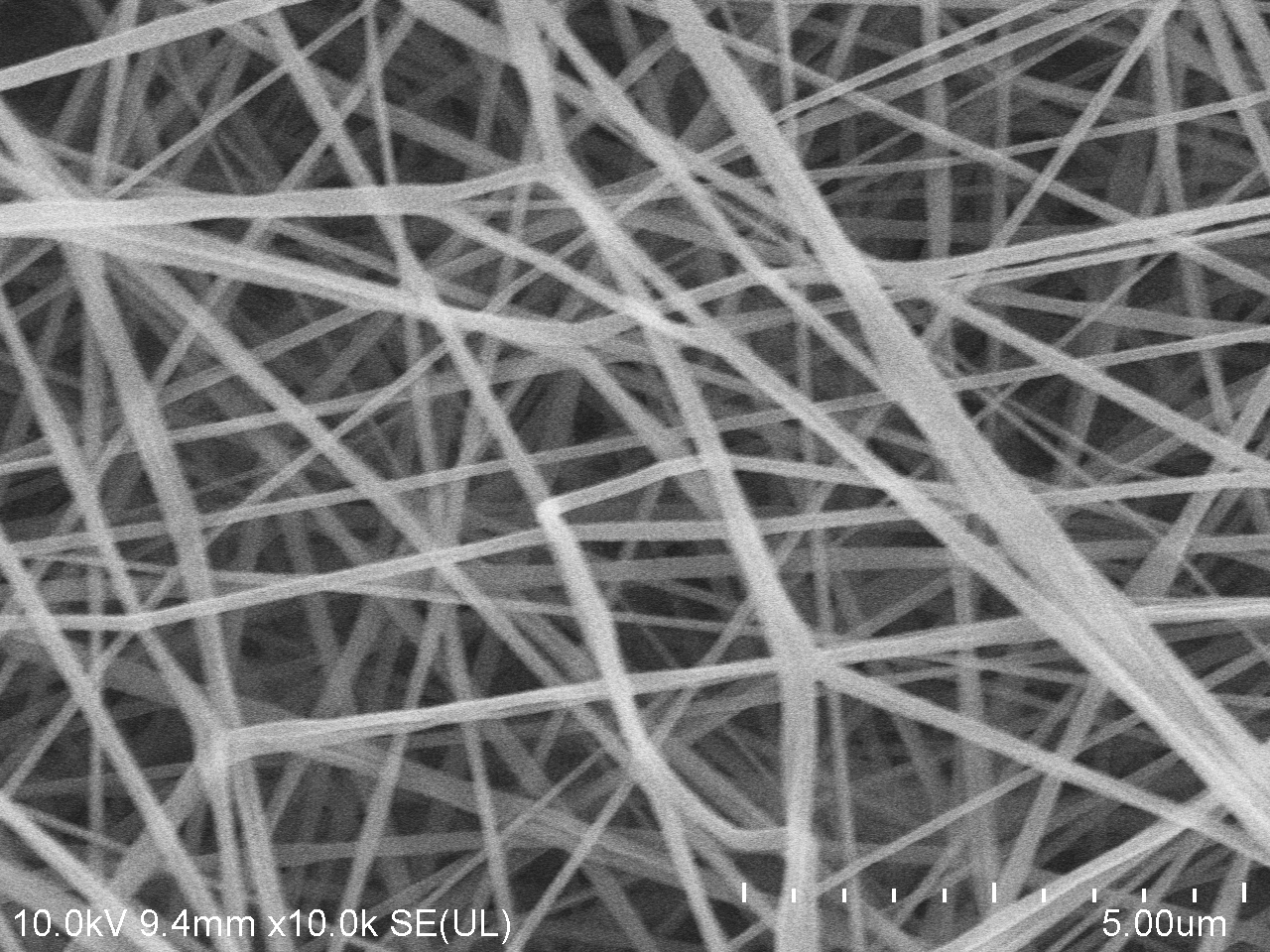

Supplement: Supplementary file 1 [file DataSheet1.ZIP › Fig2 SEM/1_i003.tif]

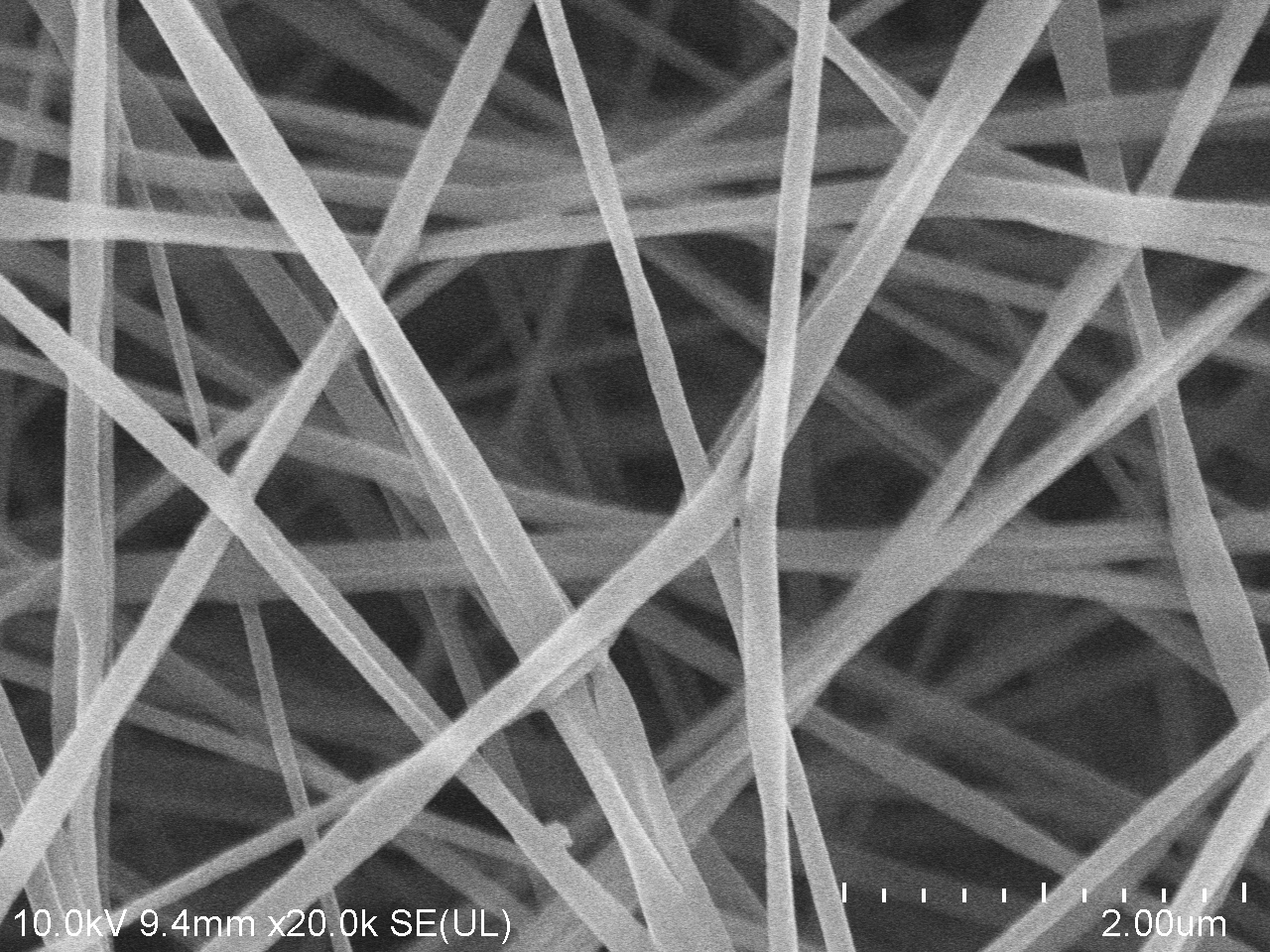

Supplement: Supplementary file 1 [file DataSheet1.ZIP › Fig2 SEM/1_i005.tif]

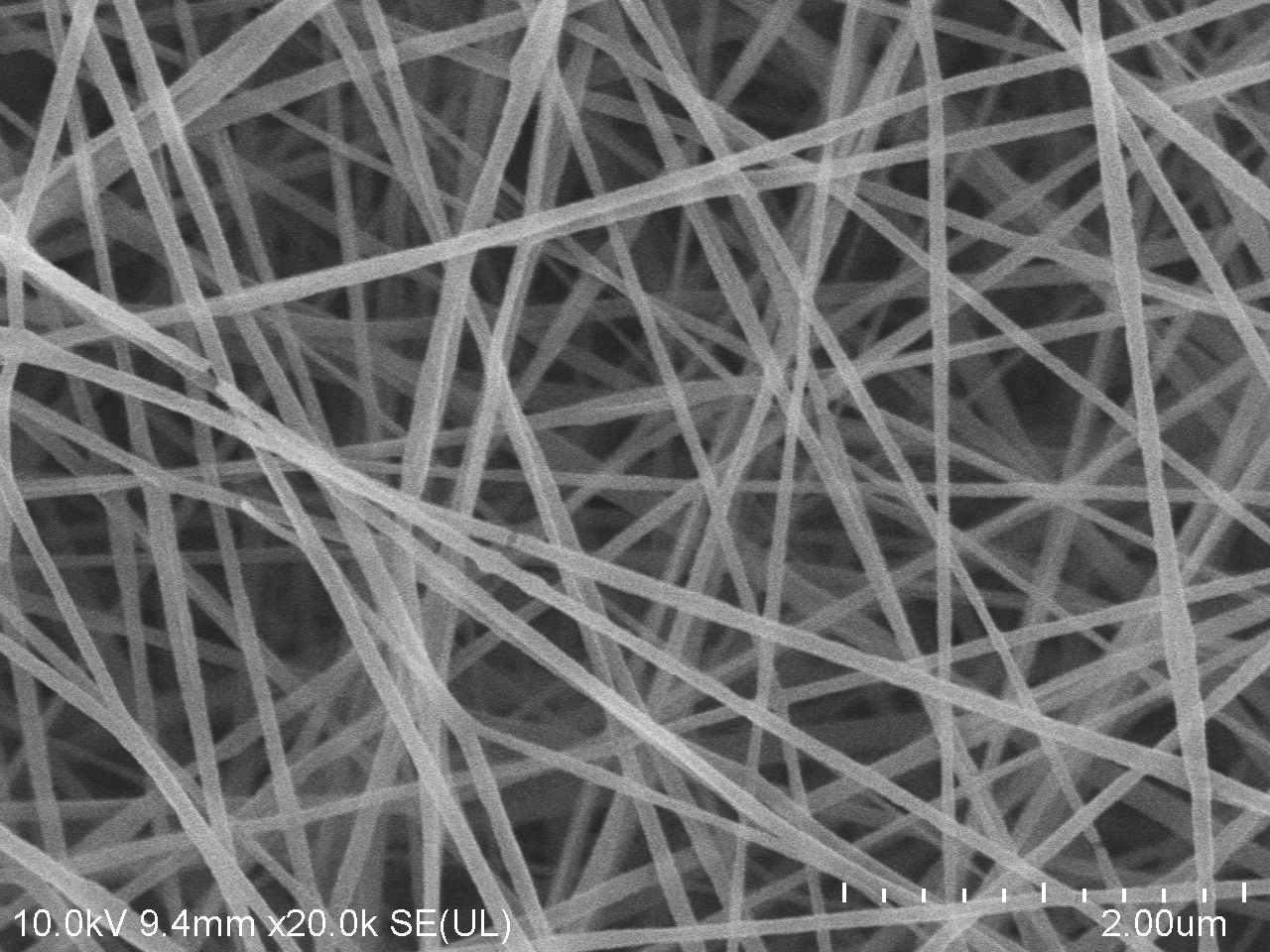

Supplement: Supplementary file 1 [file DataSheet1.ZIP › Fig2 SEM/2_i006.tif]

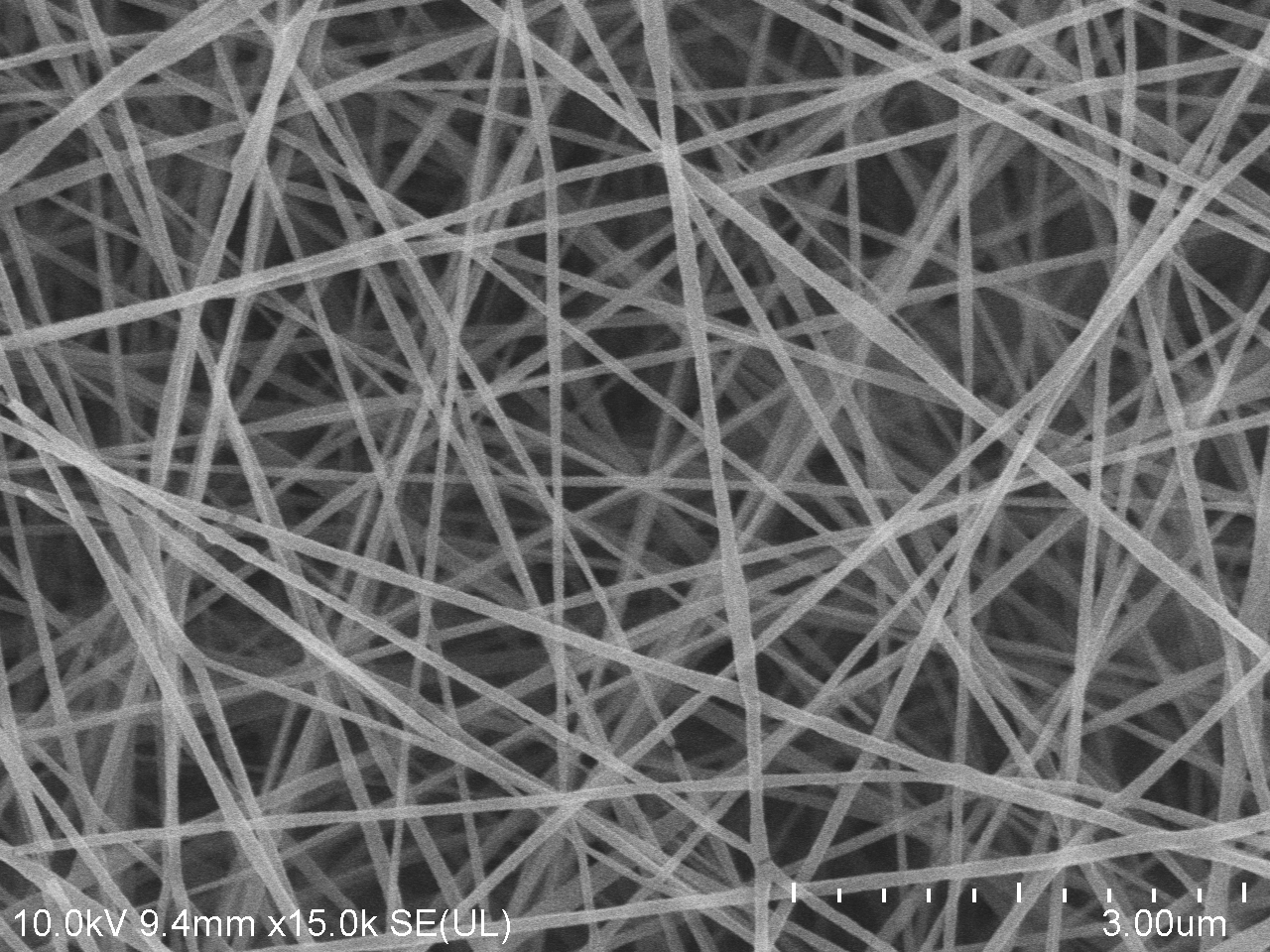

Supplement: Supplementary file 1 [file DataSheet1.ZIP › Fig2 SEM/2_i008.tif]

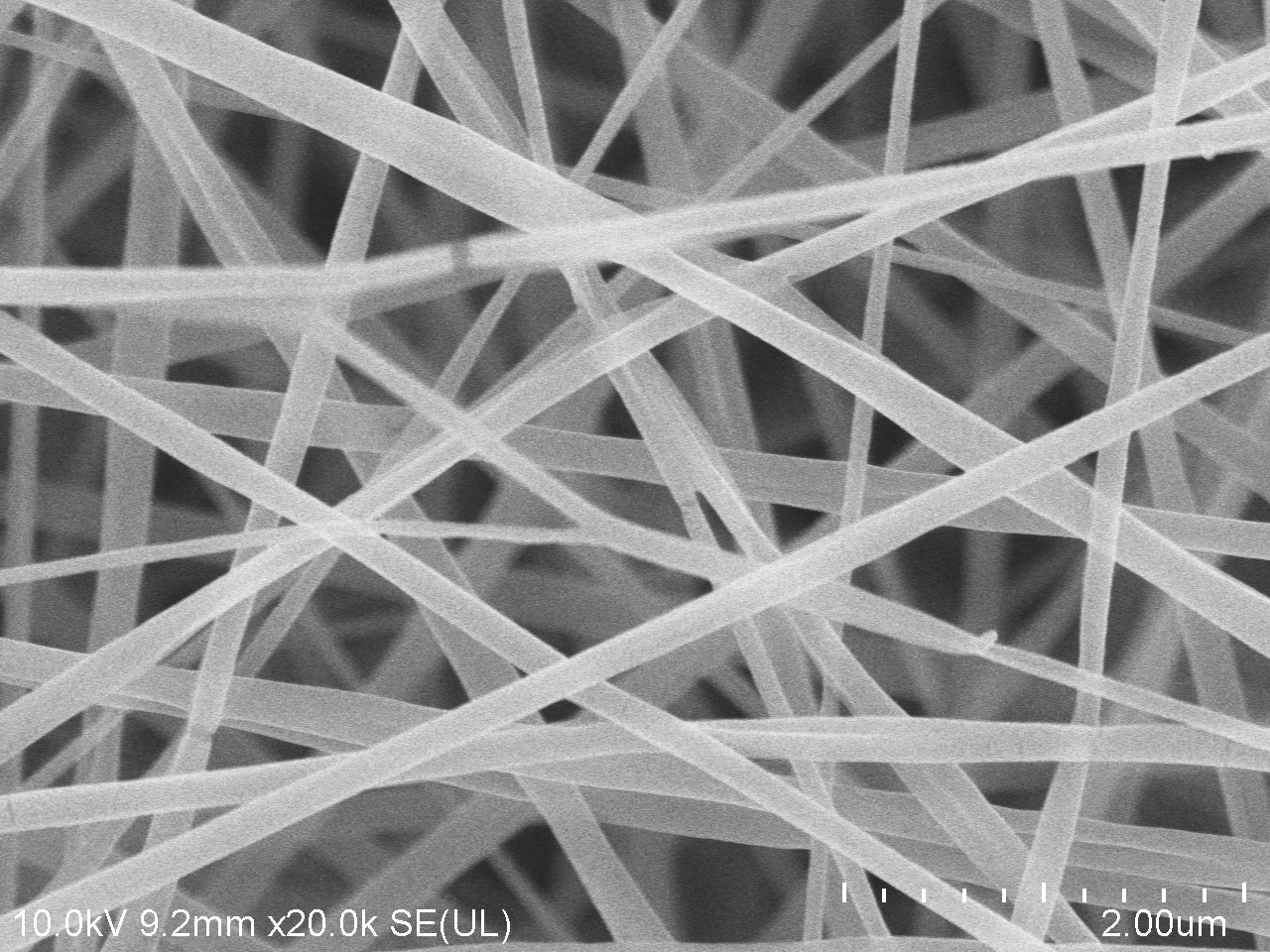

Supplement: Supplementary file 1 [file DataSheet1.ZIP › Fig2 SEM/3_i015.tif]

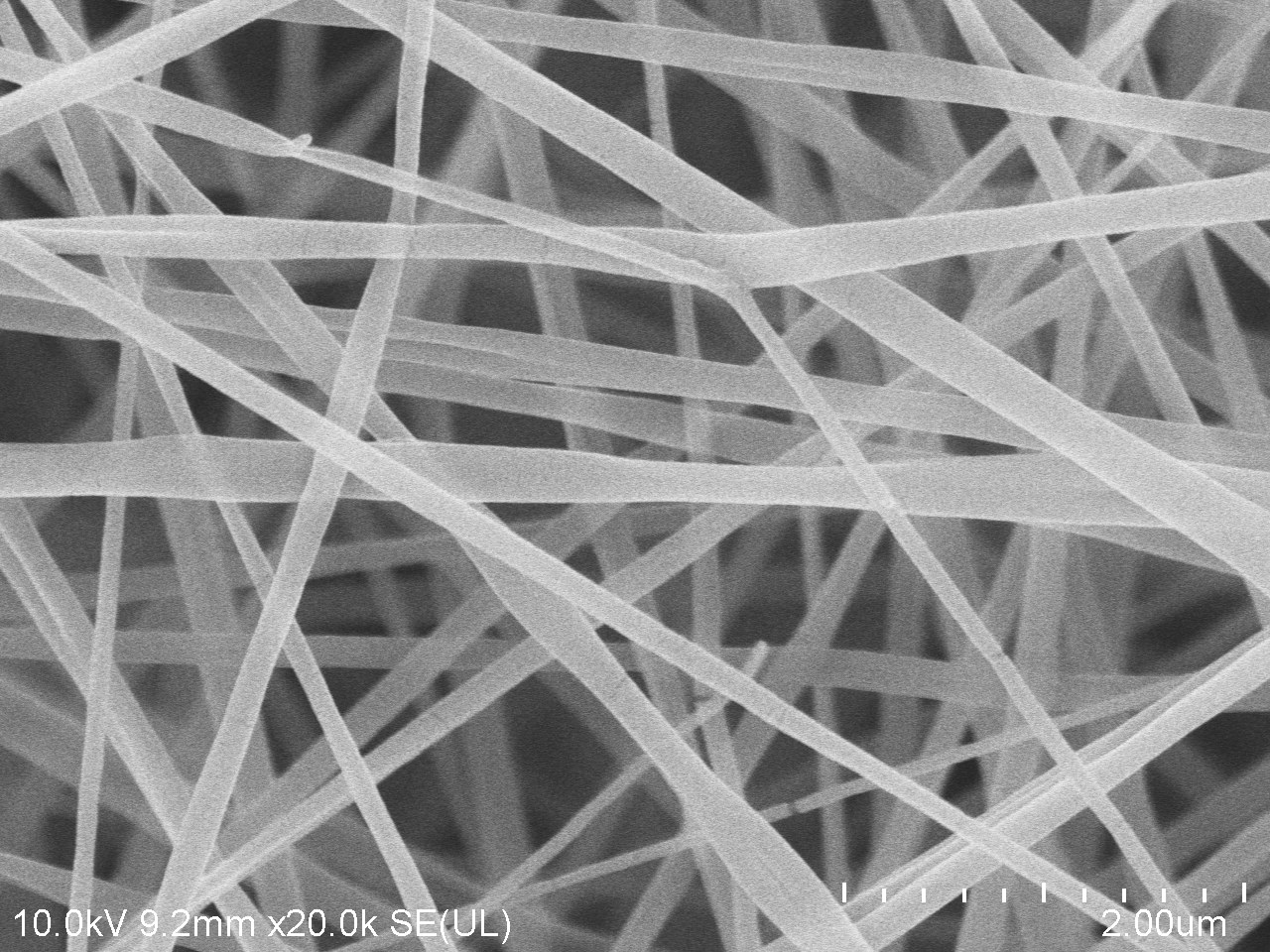

Supplement: Supplementary file 1 [file DataSheet1.ZIP › Fig2 SEM/3_i016.tif]

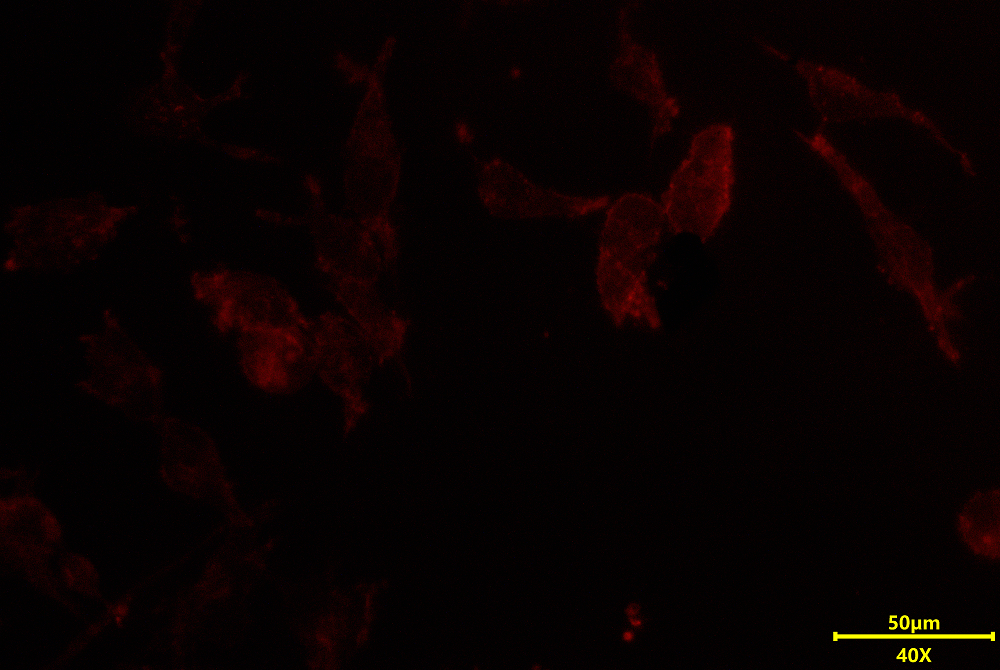

Supplement: Supplementary file 1 [file DataSheet1.ZIP › Fig5 fluorescence image/1#-1.tif]

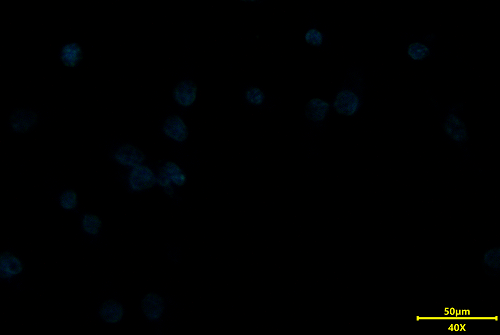

Supplement: Supplementary file 1 [file DataSheet1.ZIP › Fig5 fluorescence image/1#-2.tif]

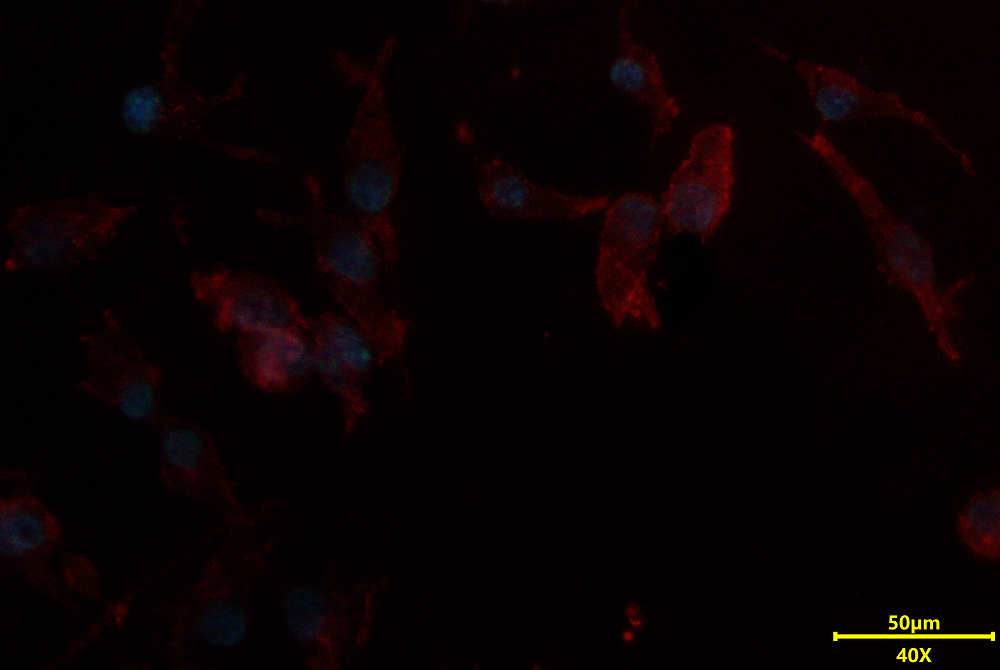

Supplement: Supplementary file 1 [file DataSheet1.ZIP › Fig5 fluorescence image/1#-3.tif]

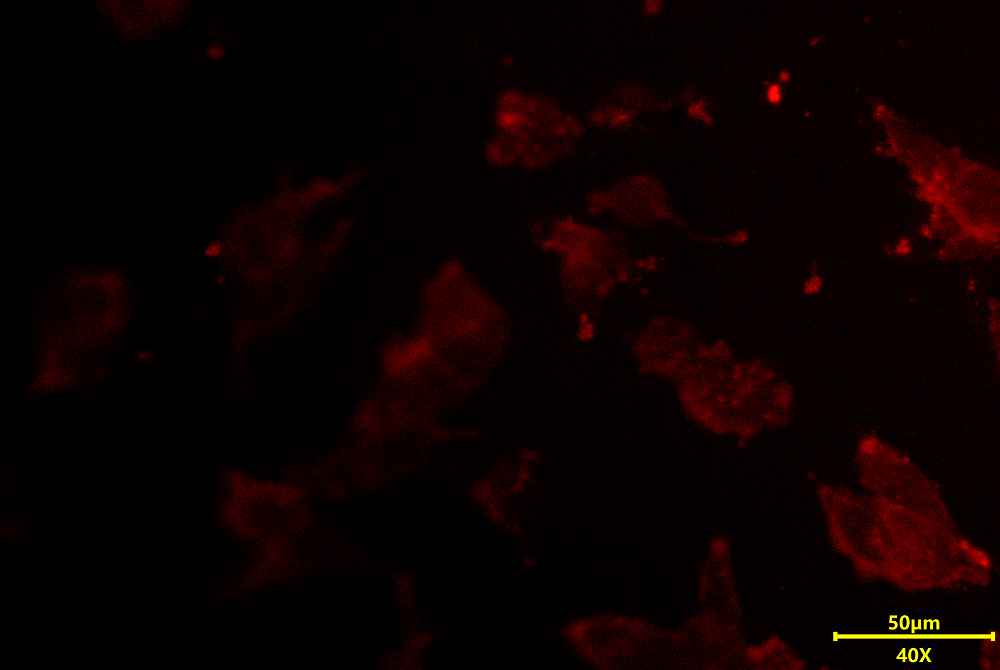

Supplement: Supplementary file 1 [file DataSheet1.ZIP › Fig5 fluorescence image/2#-1.tif]

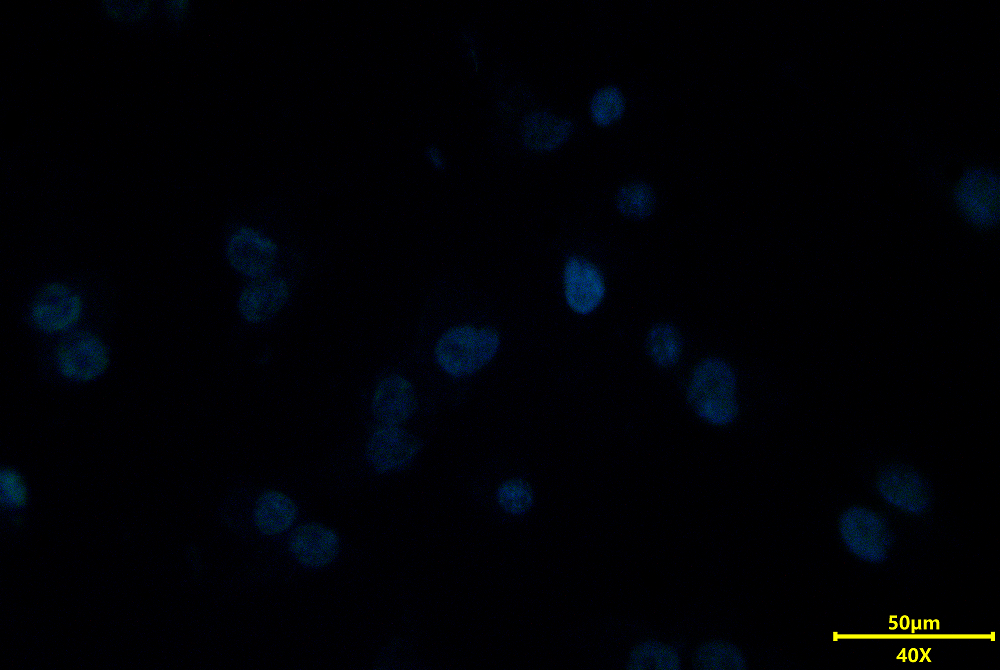

Supplement: Supplementary file 1 [file DataSheet1.ZIP › Fig5 fluorescence image/2#-2.tif]

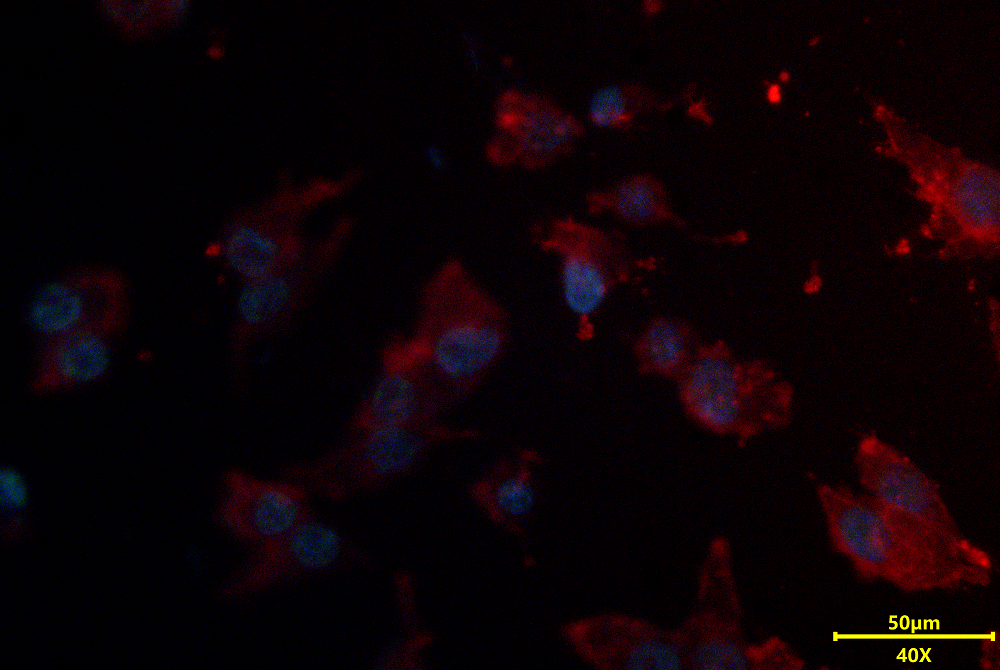

Supplement: Supplementary file 1 [file DataSheet1.ZIP › Fig5 fluorescence image/2#-3.tif]

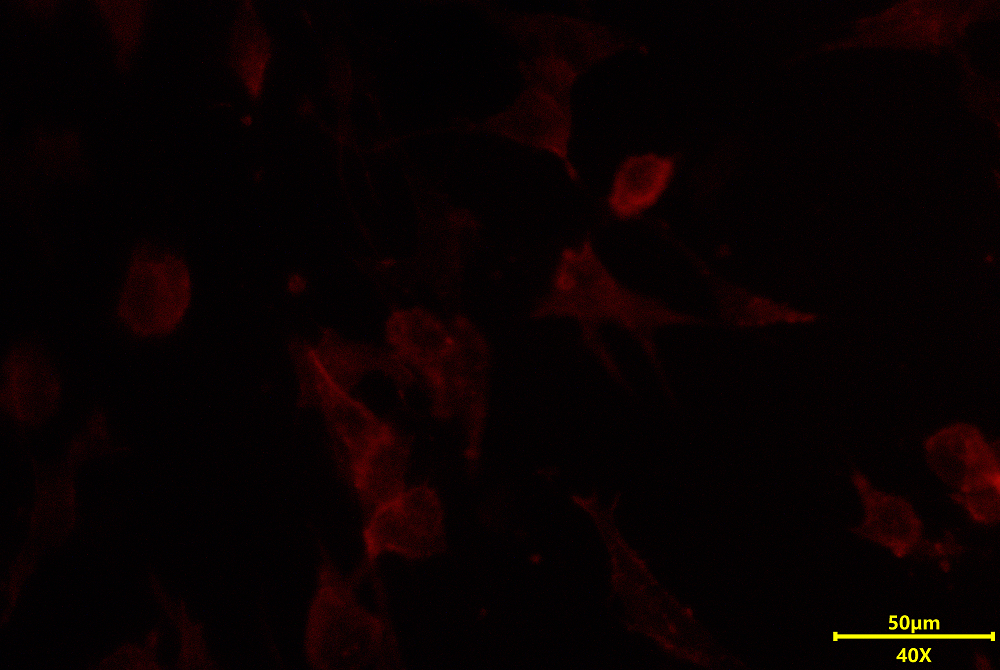

Supplement: Supplementary file 1 [file DataSheet1.ZIP › Fig5 fluorescence image/3#-1.tif]

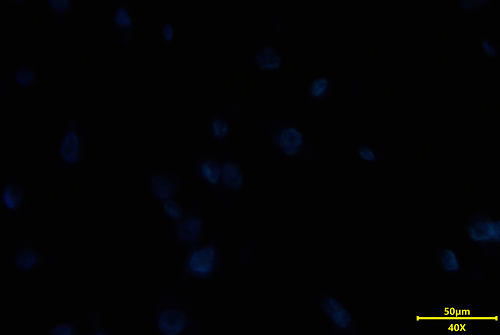

Supplement: Supplementary file 1 [file DataSheet1.ZIP › Fig5 fluorescence image/3#-2.tif]

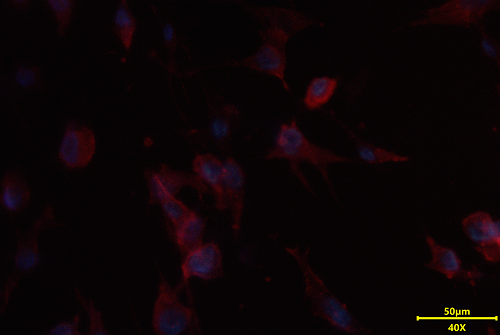

Supplement: Supplementary file 1 [file DataSheet1.ZIP › Fig5 fluorescence image/3#-3.tif]

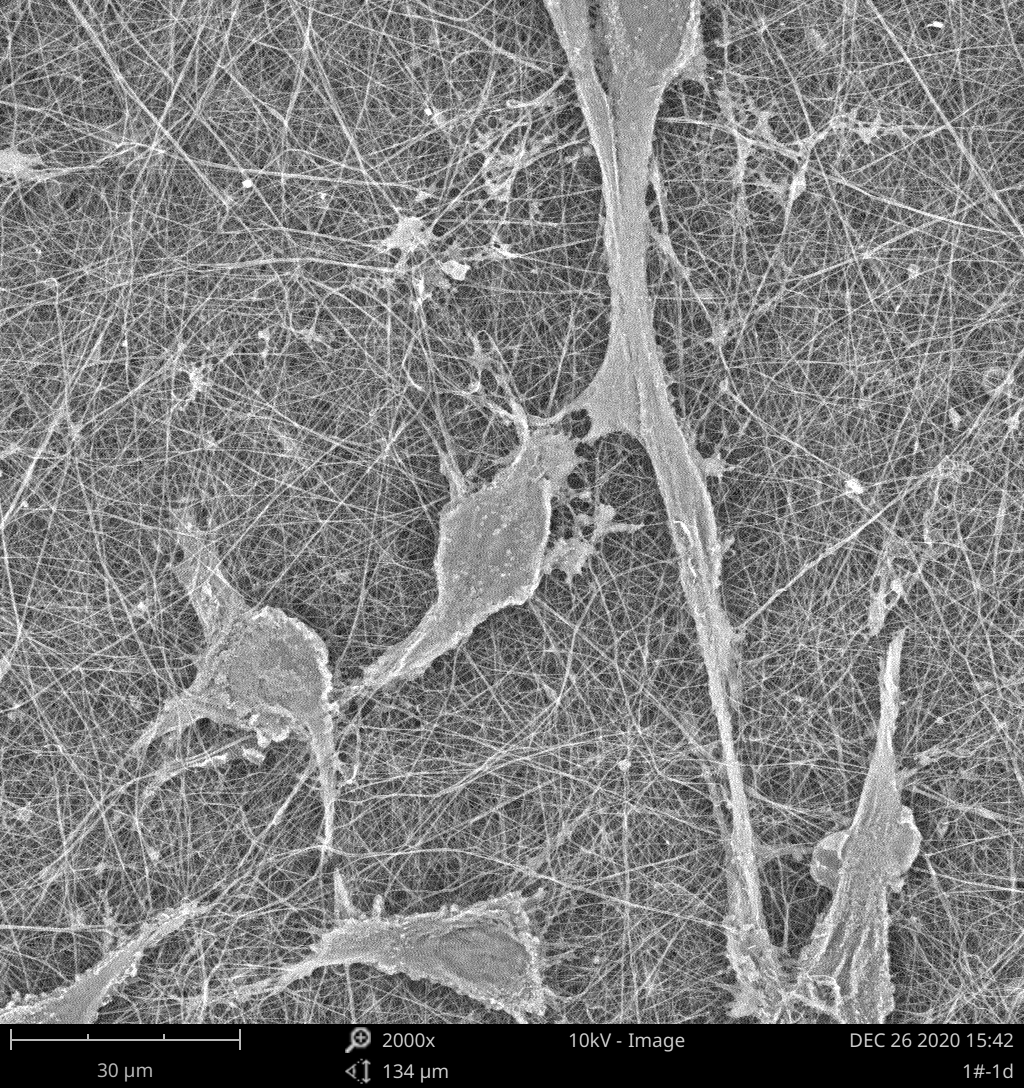

Supplement: Supplementary file 1 [file DataSheet1.ZIP › Fig6 cell SEM/1#-0001.tiff]

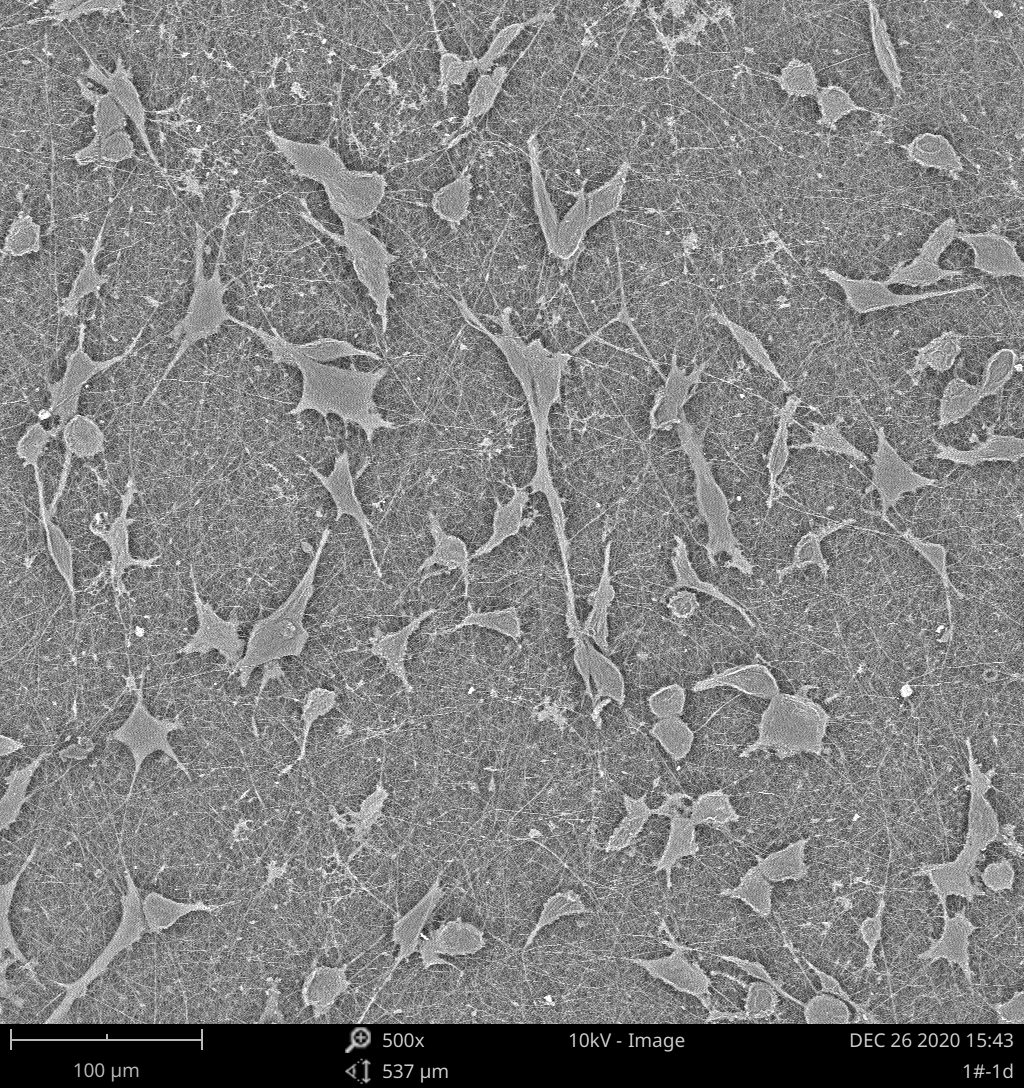

Supplement: Supplementary file 1 [file DataSheet1.ZIP › Fig6 cell SEM/1#-0002.tiff]

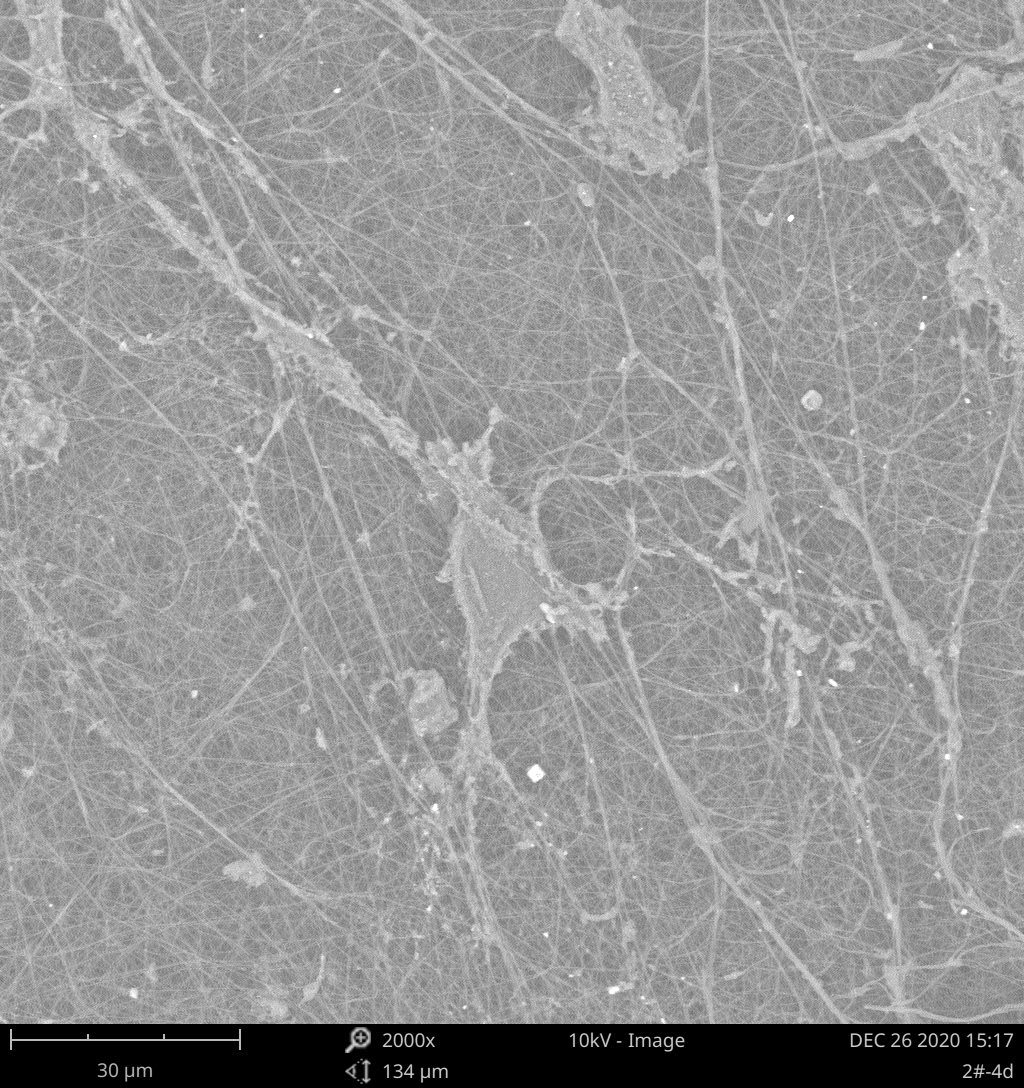

Supplement: Supplementary file 1 [file DataSheet1.ZIP › Fig6 cell SEM/2#-0001.tiff]

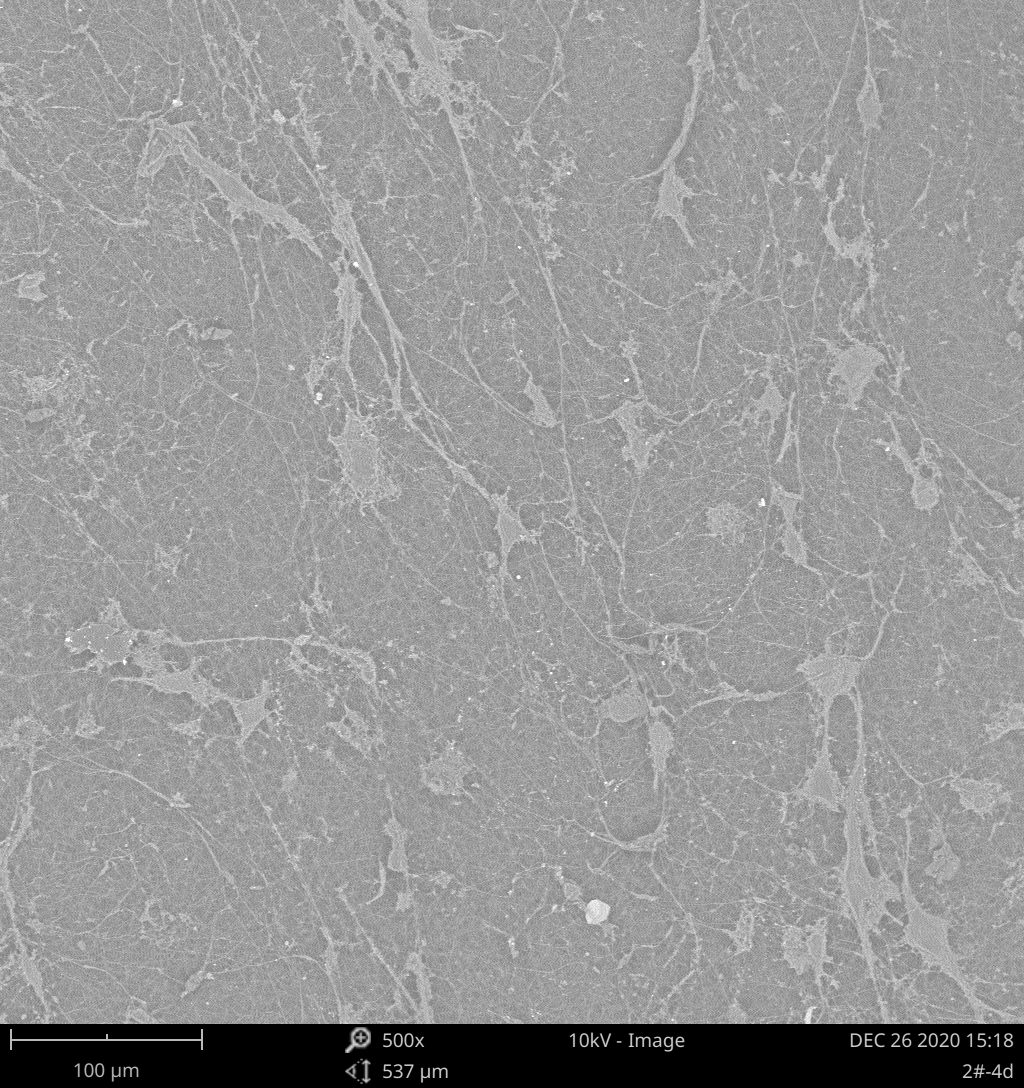

Supplement: Supplementary file 1 [file DataSheet1.ZIP › Fig6 cell SEM/2#-0002.tiff]

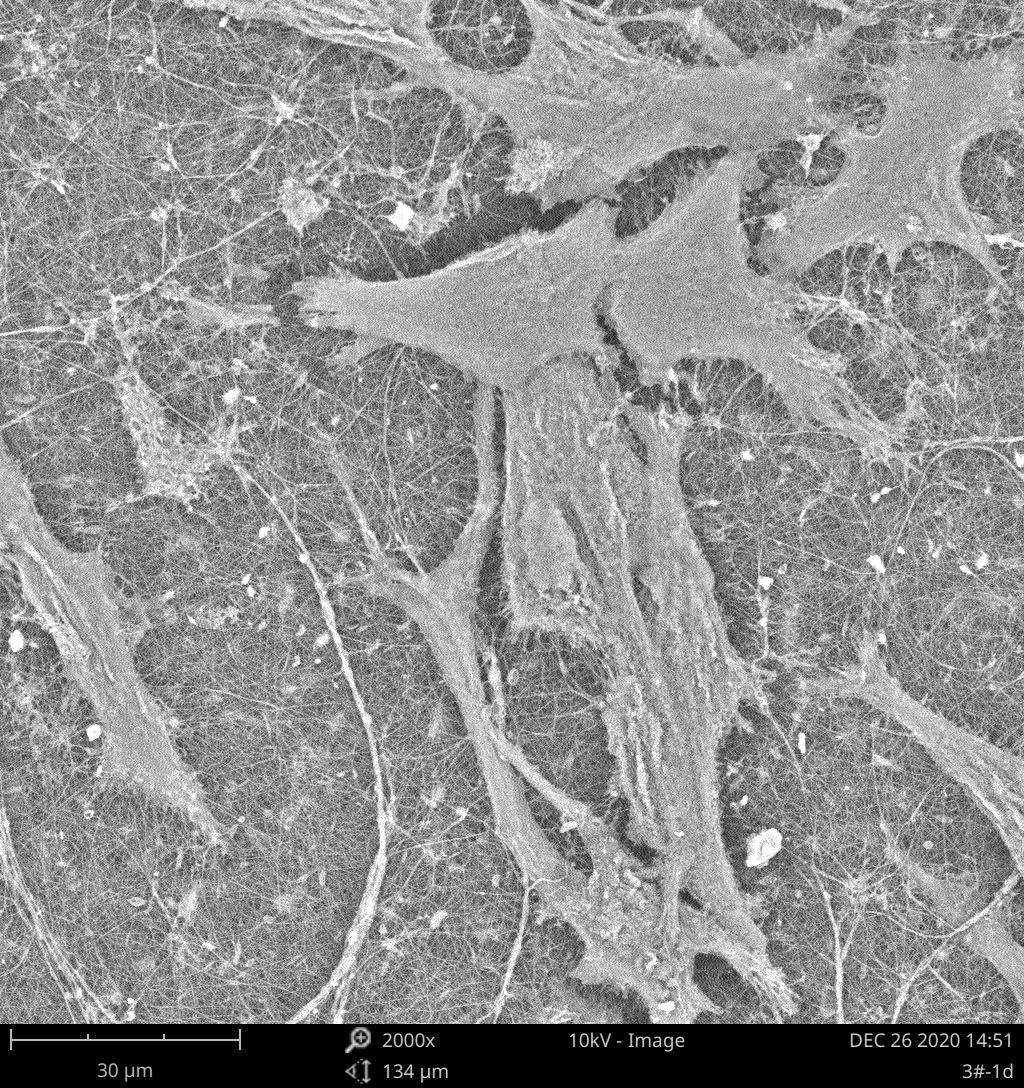

Supplement: Supplementary file 1 [file DataSheet1.ZIP › Fig6 cell SEM/3#-0001.tiff]

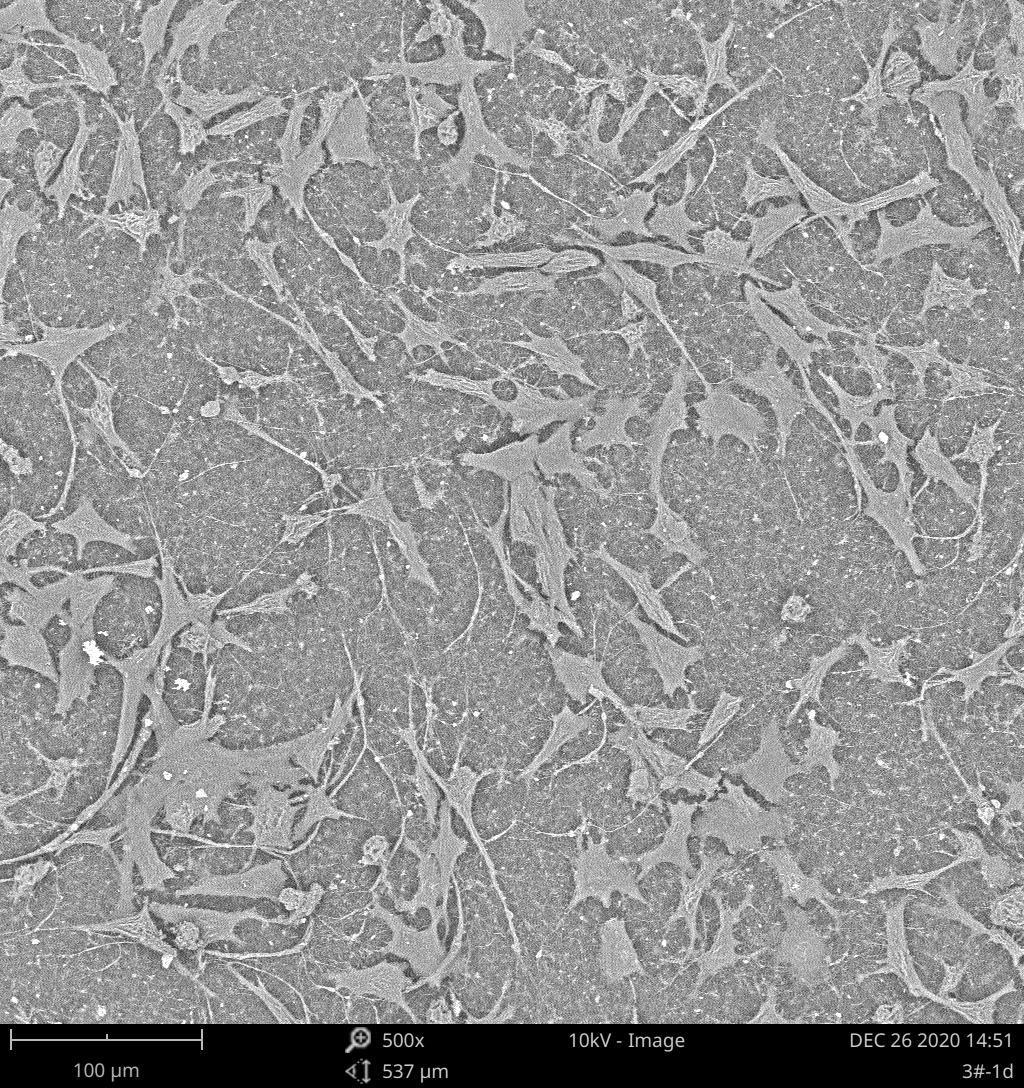

Supplement: Supplementary file 1 [file DataSheet1.ZIP › Fig6 cell SEM/3#-0002.tiff]

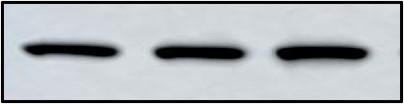

Supplement: Supplementary file 1 [file DataSheet1.ZIP › Fig6 West blot/actin-day 1.jpg]

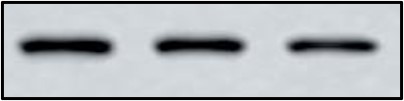

Supplement: Supplementary file 1 [file DataSheet1.ZIP › Fig6 West blot/actin-day 4.jpg]

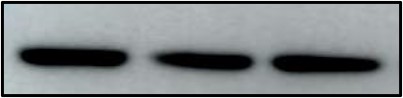

Supplement: Supplementary file 1 [file DataSheet1.ZIP › Fig6 West blot/actin-day 7.jpg]

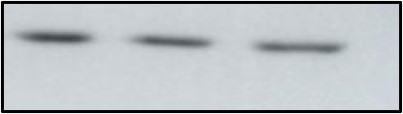

Supplement: Supplementary file 1 [file DataSheet1.ZIP › Fig6 West blot/OCN-day 1.jpg]

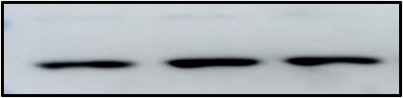

Supplement: Supplementary file 1 [file DataSheet1.ZIP › Fig6 West blot/OCN-day 4.jpg]

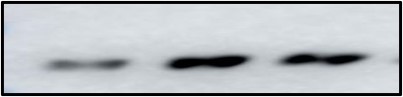

Supplement: Supplementary file 1 [file DataSheet1.ZIP › Fig6 West blot/OCN-day 7.jpg]

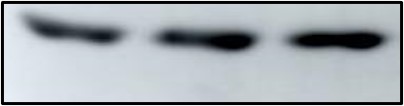

Supplement: Supplementary file 1 [file DataSheet1.ZIP › Fig6 West blot/RUNX2-day 1.jpg]

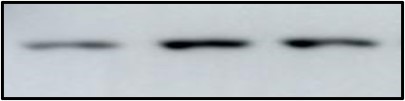

Supplement: Supplementary file 1 [file DataSheet1.ZIP › Fig6 West blot/RUNX2-day 4.jpg]

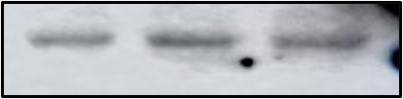

Supplement: Supplementary file 1 [file DataSheet1.ZIP › Fig6 West blot/RUNX2-day 7.jpg]
